# Nonlinear directional emission on monolayer MoS_2_ by optical orbit-orbit coupling

**DOI:** 10.1126/sciadv.aei3860

**Published:** 2026-09-30

**Authors:** Xianglong Wang, Xinghao Wang, Xueli Jiang, Shibo Xu, Zhaohui Ye, Liumiao Gao, Zhenghong Li, Zhiyuan Yan, Chaowei Wang, Yang Chen, Jiawen Li, Yanlei Hu, Dong Wu, Jincheng Ni

**Affiliations:** ^1^ Department of Precision Machinery and Precision Instrumentation, University of Science and Technology of China, Hefei 230027, China.; ^2^ Laser Institute, Qilu University of Technology (Shandong Academy of Sciences), Jinan 250353, China.; ^3^ State Key Laboratory of Analog and Mixed-Signal VLSI, Institute of Microelectronics, Faculty of Science and Technology, University of Macau, Macau SAR, China.; ^4^ State Key Laboratory of Opto-Electronic Information Acquisition and Protection Technology, Anhui University, Hefei 230601, China.

## Abstract

Controlling the direction of light emission on the subwavelength scale is a key to manipulating light and improving efficiency in optical sensing and quantum information processing. In functional metasurfaces, photonic spin angular momentum is widely used to control the propagation of light emission by geometric phase, but it is challenging to accomplish using monolayer materials because of lacking optical resonance in atomic thickness. Furthermore, the manipulation of the optical geometric phase based on spin angular momentum beams typically offers only two tunable states. Here, we report optical control of free-space routing of second harmonic generation in monolayer MoS_2_ assisted by photonic orbital angular momenta. The experimental directionality of the second harmonic light can reach 0.84. Moreover, we can dynamically route the second harmonic generation signal on the engineered monolayer MoS_2_ with four directions, showing high-order information encryption. Our findings thus provide great flexibility and more degrees of freedom in nonlinear optical modulation, integrated photonics, and dynamic flat optics.

## INTRODUCTION

Controlling over the directional propagation and emission properties of light is the key to modulating the flow of light and improving efficiency in optical communication and quantum information processing (*1*, *2*). To control the directional emission of light waves, it normally requires appropriate light field regulation methods (*3*). Recently, it was reported that the directionality of emitted light could even be realized with specific nanostructures such as optical microcavities (*4*), waveguides (*5*–*9*), metasurfaces (*10*–*12*) and photonic crystals (*13*, *14*). Moreover, by reducing the dimension of nanostructures to the atomic scale, the atomic devices show great potential for nano-optics and integrated photonic systems (*15*). However, it is challenging to dynamically shape optical wavefront on atomic structures mainly because of the lack of optical resonant phases.

Two-dimensional (2D) materials especially 2D transition metal dichalcogenides (TMDCs) have attracted extensive attention due to their excellent optoelectronic (*16*–*19*), catalytic (*20*, *21*), and mechanical properties (*22*, *23*). Monolayer TMDCs, such as Group VIB (MoS_2_, WS_2_, MoSe_2_, WSe_2_) are direct-bandgap semiconductors with high photoluminescence quantum yields, and their valley polarization can be optically excited and modulated due to unique valley-optical selection rules (*24*, *25*). Furthermore, their thin atomic thickness and natural inversion-symmetry-breaking properties make them have large nonlinear susceptibility under the premise of relaxing phase matching (*26*). A monolayer of atoms represents the ultimate miniaturization of optical device thickness, and the exceptional optoelectronic properties of TMDCs have driven their utilization in the development of atomically thin optoelectronic devices (*15*, *27*–*29*). However, tunable directional two-dimensional material-based optical devices using photonic spin-orbit coupling typically require the assistance of metasurfaces (*1*–*3*, *11*–*13*), photonic crystals (*4*, *14*), or external fields (*30*), which results in the device thickness still being more than 100 nm (Fig. 1A). Furthermore, most spin-dependent devices only have two tunable states, which limits the degrees of freedom and multidimensional control.

**Fig. 1.**
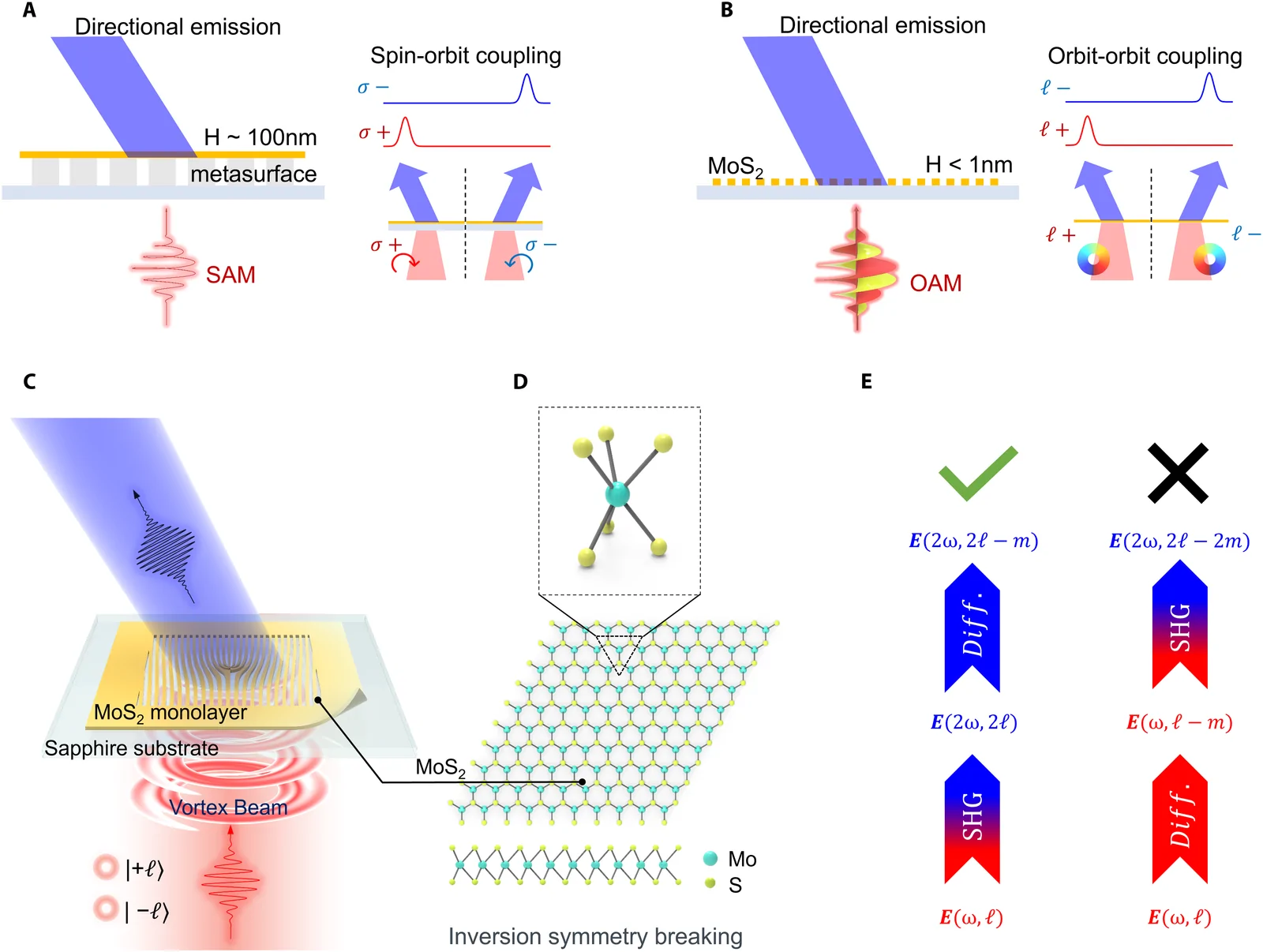
Schematic diagram of free-space routing of SH light controlled by orbit-orbit coupling. (**A**) Directional SH emission on metasurfaces with optical resonances controlled by photonic SAM. The metasurfaces, providing geometric phase for circularly polarized light, normally possess a thickness of H > 100 nm. The directional emission of SH waves can be achieved by manipulating the sign of SAM, showing spin-orbit coupling. (**B**) In our work, dynamically directional SH emission can be achieved on monolayer MoS_2_ at the thickness of H < 1 nm by photonic orbit-orbit coupling. The direction of SH waves can be controlled by the sign and magnitude of incident TCs. (**C**) Schematic illustration of the principle of directional emission resulting from a vortex beam incident on an engineered monolayer MoS_2_ with fork grating. (**D**) Schematic of the atomic structure of monolayer MoS_2_, showing its intrinsic broken inversion symmetry for SH generation. (**E**) Cascaded mechanism of the SHG and nanograting diffraction in the monolayer materials. The topological charge is extracted from the -1st order beam in the momentum space.

Optical angular momentum may serve as a viable approach for switching the directionality of photon emission in atomic-layer devices. In addition to spin angular momenta (SAM), vortex beams possess orbital angular momenta (OAM), which is an integer multiple of the reduced Planck constant ℏ (*31*, *32*). In particular, OAM beams attach great interest to free-space optical communications due to their theoretically infinite number of orthogonal topological states (*33*–*36*). Recently, the generation of nonlinear vortex beams based on 2D materials has been studied (*37*, *38*), but controlling the nonlinear directional emission by OAM beams remains unexplored.

Here, we have theoretically and experimentally demonstrated an atomic-scale photonic device based on a 2D material. Distinct from the spin-orbit coupling, structured light with OAM offers a viable solution to overcome the thickness restriction (Fig. 1B). The directionality of the second harmonic generation (SHG) from monolayer TMDCs in free space can be controlled by manipulating the sign of the topological charge (TC) of the incident vortex beam. As shown in Fig. 1C, our structures consist solely of a monolayer material on a sapphire substrate. We demonstrate a significant intensity asymmetry between the positive and negative first-order diffracted second-harmonic (SH) beams in free space by incident OAM beam with TC = ℓ. The experimental directionality of SH emission in monolayer TMDC can reach 0.84 and it increases with the grating mode *m* and the TC of the incident light, approaching the theoretical limit of 1. We achieve multidimensional routing on the ultrathin structures, enhancing flexibility for future atomic-scale optoelectronic devices and planar optics.

## RESULTS

### The mechanism underlying directional emission controlled by OAM

As shown in Fig. 1D, MoS_2_ exhibits honeycomb structure, which consists of a molybdenum (Mo) atomic layer sandwiched between two sulfur (S) atomic layers. There is a remarkable second-order nonlinear susceptibility χ^(2)^ owing to the inherent structural inversion symmetry breaking. It has been reported that the monolayer MoS_2_ belongs to the *D*_3h_ point group (*39*, *40*). Generally speaking, $\chi_{\mathrm{xxx}}^{\left(2\right)}=-\chi_{\mathrm{xyy}}^{\left(2\right)}=-\chi_{\mathrm{yyx}}^{\left(2\right)}=-\chi_{\mathrm{yxy}}^{\left(2\right)}=\chi^{\left(2\right)}$, where *x* and *y* refer to the armchair and zigzag directions, respectively (*41*).

Based on angular momentum conservation in the SHG, the projection of OAM onto the optical axis is conserved. Given an incident fundamental beam possessing OAM with TC = ℓ, the resulting SH field comprises photons characterized by OAM with TC = 2ℓ (*42*, *43*). Distinct from the flipping of the SAM that depends on the symmetry of the monolayer material (*41*), the doubling of the OAM can be attributed to the angular momentum conservation (see Supplementary Note 1 for details). Here, we utilized the cylindrical lens transformation method to demonstrate the topological charge of vortex beams. The number of dark stripes equals the absolute value of the topological charge, while the orientation of the stripes indicates the sign. For the second-harmonic beams generated by 2D materials, the number of stripes is doubled and the sign remains identical (fig. S2).

We fabricated computer-generated binary fork grating holograms on chemical vapor deposition (CVD) grown monolayer MoS_2_ using femtosecond laser direct writing (see Methods for details). The fork grating is formed by the superposition of a helical phase with mode *m* and a gradient phase with a period of Λ. The binary amplitude modulation of the second-order nonlinear susceptibility given by (*44*, *45*)

$$\chi_{\text{eff}}^{\left(2\right)}\left(x,y\right)=\chi^{\left(2\right)}\;\left\{\frac{1}{2}+\frac{1}{2}\text{sign}\left[\text{cos}\left\{\frac{2\mathrm{\pi x}}{\Lambda }-\phi \left(x,y\right)\right\}-\text{cos}\left\{{\text{sin}}^{-1}A\left(x,y\right)\right\}\right]\right\}$$ (1)

where ϕ(x,y) and A(x,y) are the phase and amplitude profiles of desired beam wavefront in the hologram plane. The phase profile of the fork grating with mode *m* is

$$\phi \left(x,y\right)=\left\{ \begin{array}{c} m\cdot {\text{tan}}^{-1}\left(\frac{y}{x}\right)\;x\geq 0\\ m\cdot \left({\text{tan}}^{-1}\left(\;\frac{y}{x}\;\right)+\pi \right)\;x<0 \end{array}\right.$$ (2)

and the amplitude profile is A(x,y)=1. In analogy to the spin-valley locking observed in monolayer MoS_2_ (*46*), an angular momentum conservation emerges in monolayer TMDCs and other nonlinear materials when illuminated with a beam carrying OAM, resulting in the SHG beam carrying twice the TC. It has been widely reported that the SH vortex beam with TC = *m* appears at the first order when a fundamental Gaussian beam is incident upon such a fork grating. When incident by OAM beams, as shown in Fig. 1E, the sequential process of SHG and nanograting diffraction determines the resulting vortex configurations. Considering an incident vortex (ℓ) and nanograting (*m*), the diffraction-SHG path follows ℓ → ℓ − *m* → 2(ℓ − *m*), leading to a −1st-order *k*-space beam with a charge of 2ℓ − 2*m*. In contrast, the charge evolves as ℓ → 2ℓ → 2ℓ − *m* by following the SHG-diffraction path. Intriguingly, although both the SHG process and nanograting modulation are generated on the same monolayer 2D materials, the final optical phenomenon shows that the SHG process precede the diffraction process. It is reasonable because the SHG process can only occur in the ultrathin monolayer materials. Consequently, the nonlinear polarization wave is inherently patterned according to the nanograting geometry at the moment of its inception. These spatially modulated polarization sources subsequently radiate into the far field, where they undergo coherent superposition to form diffraction orders. Furthermore, the TC of the *k*-th diffraction order is given by 2ℓ + *k* · *m* (see further theoretical derivations in Supplementary Note 2). First, the nonlinear process occurs when the beam is incident, which effectively doubles the TC of the incident beam. Subsequently, the SHG beam interacts linearly with the nanograting, resulting in diffraction modulation. Particularly, if *m* = 2ℓ, a bright spot will be observed in the -1st order diffraction, while a significantly larger ring appears in the opposite diffraction order, thus achieving a substantial intensity contrast between the two diffraction orders and enabling directional emission from the ultrathin material, as shown in Fig. 1B. Moreover, for materials that support of *n*th-order high-harmonic generation, a generalized relation can be derived: ℓ_nω_ = *n*ℓ_ω_ + *km*, where *n* is the harmonic order, ℓ_ω_ is the OAM of the pump beam. Directional emission can thus be similarly achieved by combining the nonlinear frequency conversion with the phase modulation from the structured beam. In our experiments, the directionality of the emission changes when the sign of the topological charge of the incident vortex beam is altered. The angle of directional routing is governed by the grating period. According to the Raman-Nath diffraction regime, the emission angle in ultrathin materials is (*47*)

$$\theta_{\text{SHG}}={\text{sin}}^{-1}\left(\frac{\lambda_{\text{SHG}}}{\Lambda }\right)$$ (3)

where λ_SHG_ is the SH wavelength. We present a simple method for achieving directional beam routing on ultrathin materials. When the material thickness is reduced to monolayer, its linear light–matter interaction becomes very weak. In our experiments, the monolayer MoS_2_ has a thickness of only ∼0.7 nm, which is far smaller than the operating wavelength of femtosecond laser light (∼800 nm). Consequently, the light-matter interaction path is extremely short, resulting in a very small linear optical response—typically, the absorption of monolayer MoS_2_ is less than 2% at the wavelength of ∼800 nm (*48*). In our experiments, no obvious directionality can be measured in the linear processes on the ultrathick device (fig. S3). However, in the nonlinear regime, we can observe a clear directionality of second-harmonic diffraction on the patterned fork grating.

### Optical setup and second-harmonic detection

The optical setup is shown in Fig. 2A. The laser system emits stable pulsed laser light with a central wavelength of 796 nm and a pulse width of 75 fs. A reflective phase-only spatial light modulator (SLM) is used to generate vortex beams with varying topological charges. The vortex beam is focused onto the MoS_2_ with a 50× objective, and the generated second harmonic signal is collected. A low-pass filter is placed in the collection path to block the residual fundamental frequency beam. Finally, the SH beam is focused onto a charge coupled device (CCD). The observing *k*-space can be achieved by adding an additional Fourier lens to the optical path. Figure 2B shows triangular monolayer MoS_2_ grown on a sapphire substrate using CVD.

**Fig. 2.**
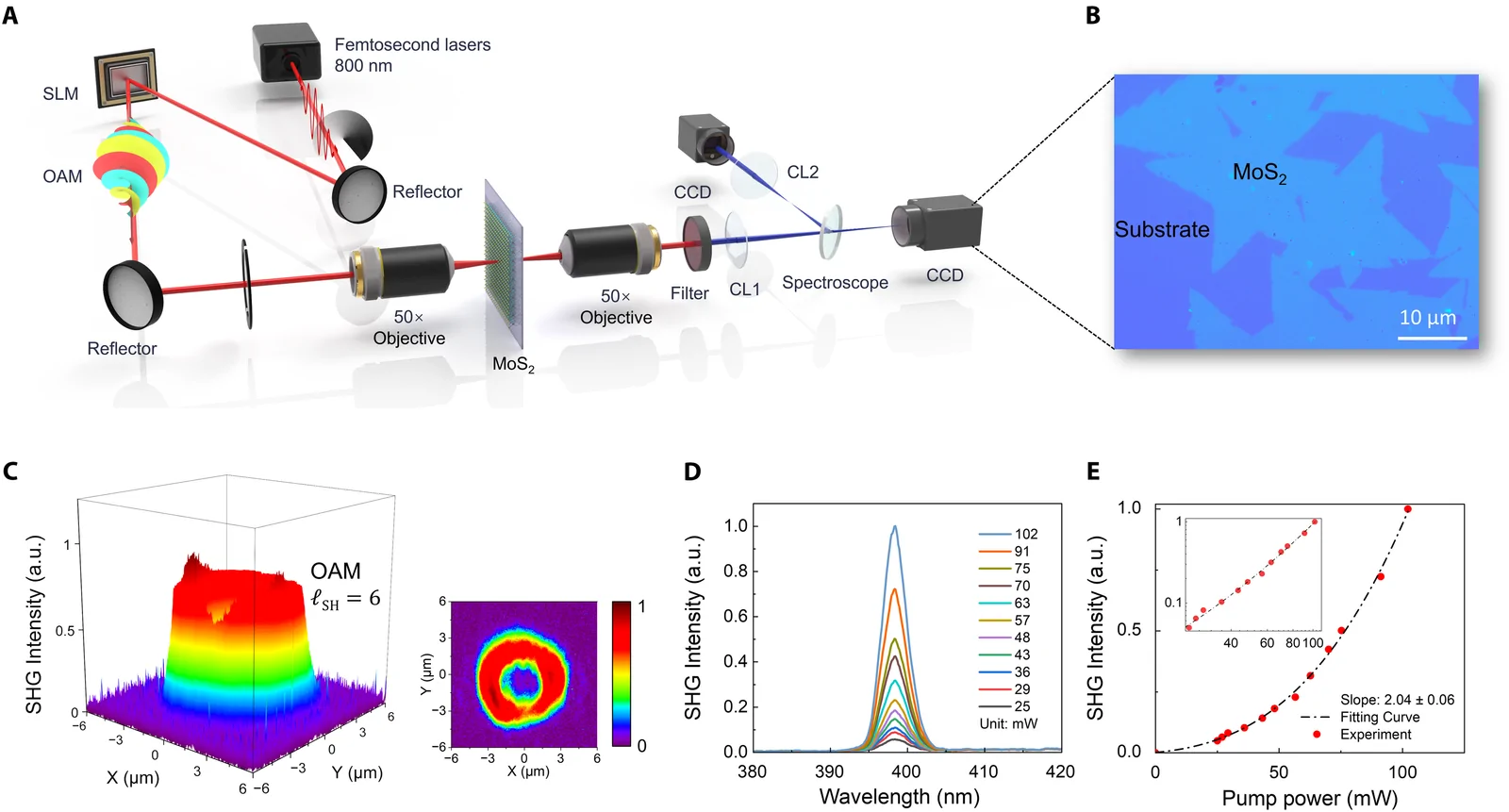
Experimental optical setup and SH generation results. (**A**) Optical setup. A femtosecond laser beam is incident on a phase-only spatial light modulator (SLM) to generate vortex beams with different topological charges. Then the beams are focused onto the engineered nanostructures using a 50× objective. The diffracted far-field information is collected by a CCD camera. A filter is used to remove the fundamental frequency, and an additional Fourier lens is used to observe the momentum-space information. CL: convex lens. (**B**) Optical microscopy image of a monolayer MoS_2_. (**C**) Schematic of the normalized SH structured light field of a vortex beam with a topological charge of ℓ = 6. (**D**) Normalized SH spectra of monolayer MoS_2_ at different pump powers, showing a corresponding central wavelength of 398 nm. (**E**) Normalized power-dependent SH intensity. The linearly fitted slope value is 2.04 (inset), confirming the quadratic nature of the SH process.

As shown in Fig. 2C, an optical microscope photograph of the size and morphology of the SH vortex beam with ℓ = 6 is generated when the incident fundamental vortex beam with ℓ = 3. In our optical setup, the SH vortex beam exhibits a well-defined donut-shaped intensity distribution with a diameter of ∼10 μm. Figure 2D demonstrates the spectral curve of a vortex beam with a central wavelength of 398 nm under different incident optical powers (from 25 to 102 mW). Figure 2E shows the variation of the peak intensity as a function of incident beam power, where the fitted curve is a quadratic curve and a linear fit to the log-log plot has a slope of 2.04, demonstrating the second-order nonlinear process. In the SHG in monolayer MoS_2_, the photon energy of the 796 nm fundamental excitation light is higher than the A-exciton resonance energy. Therefore, the small deviation from an expected slope of 2 may arise from experimental uncertainties or weak bandgap modulation (*49*).

### Free-space harmonic directional routing controlled by vortex beams

For unidirectional routing of nonlinear photons, our ultrathin device includes a monolayer binary forked grating MoS_2_ on sapphire substrate incident by fundamental OAM with ℓ = 3 (Fig. 3A). A nanograting structure with *m* = 6 was fabricated on a monolayer MoS_2_ film by femtosecond laser direct writing technique. The grating period of Λ = 800 nm is employed and the structured area is confined to a 15 × 15 μm^2^ region. According to Eq. 3, the harmonic diffraction angle is 30°, corresponding to *k*_*x*_/*k* ≈ 0.5. To demonstrate whether material removal affects the SHG efficiency of the monolayer material, we measured the harmonic generation intensity of the monolayer before material processing and after engineered (Fig. 3B). The fork grating exhibits approximately 57% of the harmonic generation efficiency compared to the complete material. The 57% retention, which corresponds well with the grating duty cycle, indicates that our fabrication process does not drastically impact the monolayer harmonic generation efficiency. Imperfect fabrication can lead to a small deviation of the actual duty cycle from the designed value of 0.5, which will induce a slight reduction in the overall optical intensity. However, the imperfections do not disrupt the wavefront matching condition or modify the orbital angular momentum selection rule. Consequently, the directionality of the nonlinear emission is robust in our experiments.

**Fig. 3.**
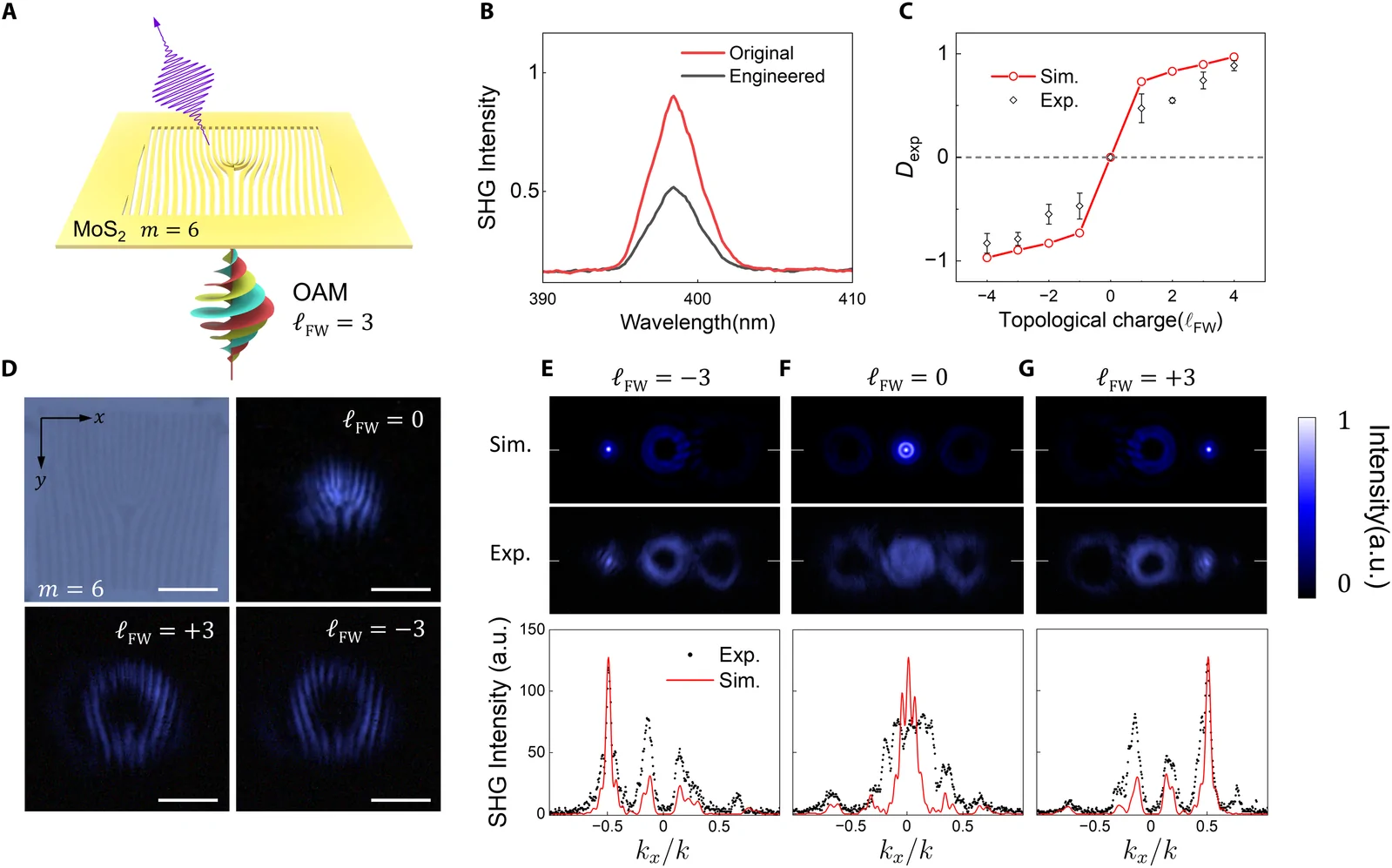
Simulated and experimental demonstration of OAM-controlled SH free-space routing. (**A**) Schematic diagram of a vortex beam with ℓ = 3 incident on the fork nanograting with *m* = 6. (**B**) SHG spectra excited on monolayer MoS_2_ with/without engineered nanostructure. Approximately 57% of the intensity is retained after processing, which is comparable to the unprocessed area. (**C**) Relationship between directionality and the TC of the incident vortex beam, where the structural mode *m* is equal to 2ℓ_FW_. (**D**) Optical micrograph of the *m* = 6 fork nanograting. Dark-field optical micrographs with varying incident TC. ℓ_FW_ = −3, 0 and +3. Scale bars; 5 μm. (**E** to **G**) Experimental (top panel) and simulated (middle panel) SH *k*-space imaging and line cut of the intensity profiles (bottom panel) along the fork nanograting system excited by the vortex beam with ℓ_FW_ = −3 (E), 0 (F) and +3 (G), respectively.

Figure 3D demonstrates an optical image of our nanograting structure, and the polarization direction of our pump beam is along the *x*-axis of the coordinate system. Dark-field imaging demonstrates distinct beam fringes by incident beams with varying topological charge. The measured spot diameter is ∼10 μm, which is smaller than the dimensions of fabricated structures. Figure 3 (E to G) present experimental and simulated results demonstrating the control of harmonic directionality emission using vortex beams with ℓ = −3, 0, +3, respectively. In the experiment, the beams were incident normally on the center of the nanograting structure. When excited by the vortex beam with ℓ = 0, the *k*-space image revealed two rings with approximately equal intensity, which implied symmetric emission (Fig. 3F). Excited by vortex beam with ℓ = −3, we can find a strongly directed emission to the left. The emission was characterized by a high-intensity point-like spot on the left side that significantly exceeded the intensity of the zero-order spot and dwarfed the diffuse ring on the right (Fig. 3E). When the helicity of the vortex beam flips, the propagating direction of the SH emission is changed to another side, as shown in Fig. 3G. The experimentally obtained zero-order intensity is consistently lower than that predicted by simulations, which can be attributed to unavoidable deviations from the designed structure during the fabrication process. The intensities of the point-like spots can be used to quantify the directionality of the emission. We define the experimental directionality of SH emission as $D_{\text{exp}}=\left(I_{R}-I_{L}\right)/\left(I_{R}+I_{L}\right)$, where $I_{R/L}$ is the SH intensity peak on both sides of *k*-space, respectively. The measured results are in well agreement with the simulation predictions. In the *m* = 6 forked grating structure, a directionality of up to 0.84 can be achieved by incident beams with ℓ = ±3. It is noteworthy that a substantial improvement in experimental directionality can be realized by increasing the structural mode as shown in Fig. 3C. When the structural mode is 8, an experimental directionality of 0.89 can be achieved (fig. S4). In our experiments, we employed incident vortex beams carrying OAM states with linear polarization. Theoretically, similar results can be obtained when using incident vortex beams with circular polarization, although slight differences exist in the variation of the total angular momentum of the beam (see further theoretical derivations in Supplementary Note 3).

### Free-space four-way routing for encryption/decryption communications

Furthermore, we can extend the one-dimensional fork grating to a two-dimensional fork grating array for controlling four-directional emission of the SH beam (*50*). The phase profile of the two-dimensional arrayed fork grating is obtained by superimposing the fork grating phases along the *x* and *y* axes. The second-order nonlinear susceptibility is given by the product of the grating susceptibilities in the *x* and *y* directions and it can be described as $\chi_{\text{array}}^{\left(2\right)}=\chi_{m_{x}}^{\left(2\right)}\cdot \chi_{m_{y}}^{\left(2\right)}$, where *m*_*x*_ and *m*_*y*_ represent the mode of the fork gratings in the *x* and *y* directions, respectively. Figure 4C shows an optical microscope image of the 2D fork grating array fabricated by femtosecond laser direct writing, where *m*_*x*_ = 8 and *m*_*y*_ = 4. The polarization direction of our pump beam is along the *x*-axis of the coordinate system. The effective area of the 2D patterned structure is smaller than that of the 1D structure, but the experimentally measured SHG intensity of the 2D grating can still reach approximately 22% of the intensity of the original material (fig. S7). In accordance with the aforementioned arguments, when the TCs of the incident vortex beams are ℓ = ±4, the directional routing in the *x*-direction can be achieved, and the directional routing in the *y*-direction can be achieved by incident vortex beams with ℓ = ±2. Figure 4 (A and B) show the obtained far-field SHG images by incident TCs of −2 and +4 upon the forked grating array. When a vortex beam with ℓ = 4 is incident, a bright spot appears in the *x*-direction. Conversely, when a vortex beam with ℓ = −2 is incident, a bright spot appears in the -*y*-direction. This implies that the sign and magnitude of the TC can directly influence the direction of the SHG signal.

**Fig. 4.**
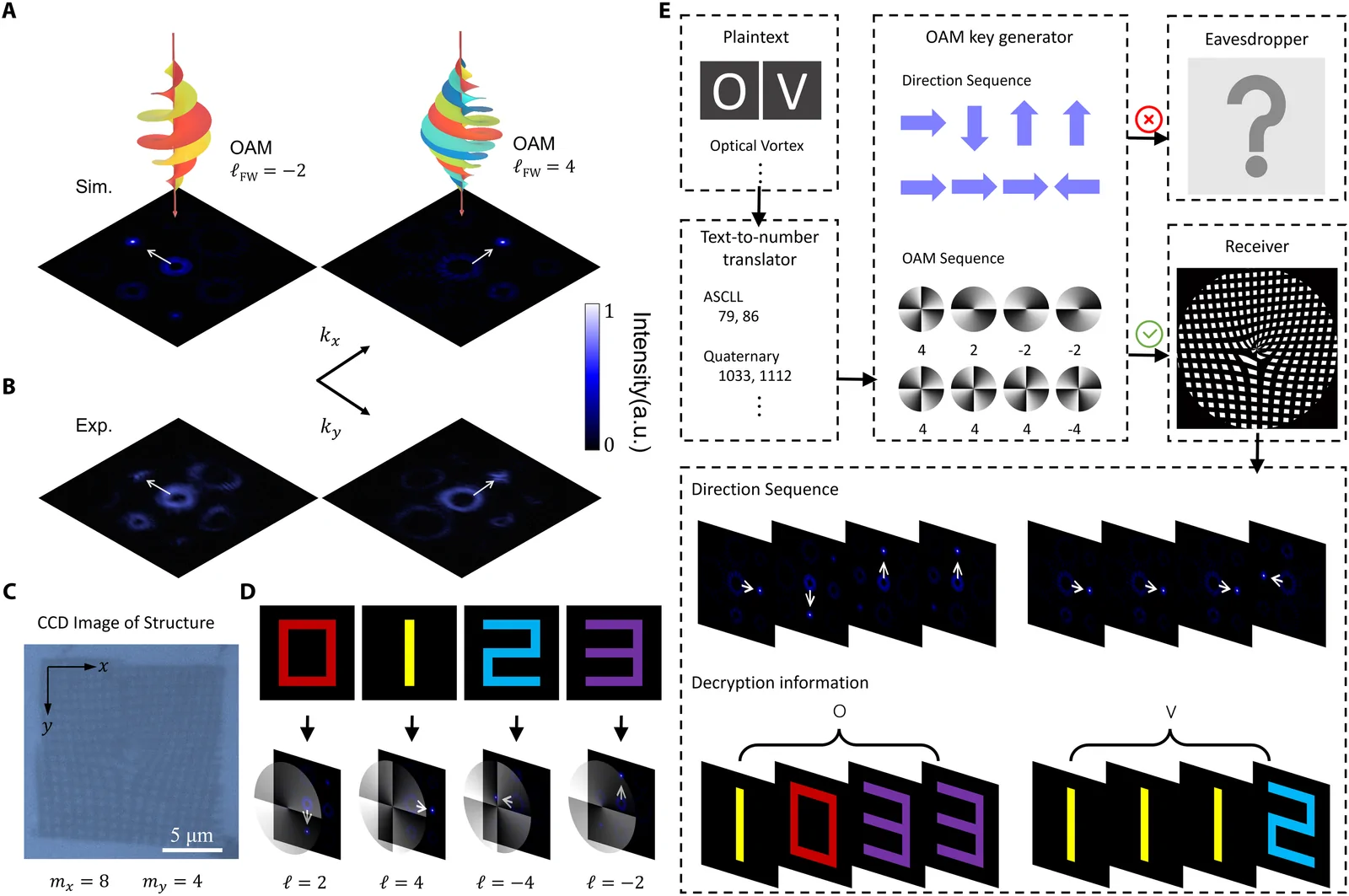
Momentum-space SH emission controlled by photonic orbit-orbit coupling. (**A** and **B**) Simulated (A) and experimental (B) *k*-space images of a 2D fork nanograting array illuminated with ℓ_FW_ = −2 and +4, respectively. (**C**) Optical microscope image of 2D engineered fork nanograting. (**D**) Designed approach is employed for quaternary encoding, where the encoded numbers correspond to the OAM states and emission direction. (**E**) Experimental methods for encryption and decryption. The ciphertext “OV” is transformed into a quaternary encoding that corresponds to a sequence of emission directions, which is then converted into an incident sequence of OAM. The OAM sequence sent in chronological order can prevent eavesdroppers and be received by the holder of the coded structure and it is restored to the correct information.

For optical communications, the unlimited OAM dimension shows more promise compared to only two states of SAM (*51*, *52*). Directionality achieved through OAM control can implement quaternary (or even higher) encoding because it has an infinite number of orthogonal states. Here, as a demonstration, we present a quaternary encryption/decryption communication method (Fig. 4, D and E). First, we map the quaternary values 0, 1, 2, and 3 to the four emission directions corresponding to four different TCs of incident vortex beams. The ciphertext “OV” which means optical vortex is transformed into a quaternary encoding that corresponds to a sequence of emission directions and then it is converted into an incident sequence of OAM sent in chronological. The incident OAM sequence is received by a receiver equipped with a specific two-dimensional fork-shaped grating array, which decodes the information into a quaternary coded sequence, ultimately yielding the correct information. As a proof of concept, we have demonstrated the encoding, transmission, and decoding of the characters “OV” as shown in movie S1.

Although we have only demonstrated an application of four-way emission, multidimensional directional emission can theoretically be achieved due to the infinite orthogonal states of vortex beams. We also demonstrated the simulated realization of higher-dimensional fork gratings. However, constrained by fabrication precision and nonlinear generation efficiency, the maximum dimension achievable in practical applications is approximately 6 (Supplementary Note 4). Combining this with the polarization-resolved and broadband characteristics of SHG in materials, future applications could realize polarization and wavelength multiplexing. Vortex beams, which possess an infinite number of orthogonal dimensions, hold great potential in optical communications (*53*, *54*).

## DISCUSSION

In summary, we have demonstrated OAM-controlled directional SH emission in free space from monolayer MoS_2_. The directionality in the MoS_2_ up to 0.84 has been experimentally recorded for ℓ = +3, which is larger than spin-coupling methods. The monolayer material used in the experiment can also be extended to other ultrathin materials capable of generating high-order harmonics. In contrast to two spin states, directional routing controlled by vortex beams allows for more directional emission and more sophisticated encoding, offering broader application prospects in optical communications. The directional asymmetry of the SH vortex beam signifies the real-space optical phase singularity introduced by the fork grating. This parallels nonlinear optical probes of momentum-space topology: in TMDs with broken time-reversal symmetry, nonlinear circular dichroism reveals Berry curvature underlying electronic band topology. In both scenarios, symmetry breaking induces a measurable asymmetric optical response, demonstrating the generality of using symmetry-induced asymmetry to characterize topological or singular structures in diverse systems (*55*). Furthermore, directional emission without spin control opens up unexplored new paradigms for the directional manipulation of other wavefields, such as other chiral light fields, electrons, sound, and even atomic waves. Orbitally oriented SH emission from ultrathin materials holds potential for practical applications in optical communications and quantum information processing (*56*, *57*). This is expected to significantly advance the development of atomic-scale optics and flat optics.

## MATERIALS AND METHODS

### Sample fabrication

The monolayer MoS_2_ films were supplied by Shenzhen Six Carbon Technology Inc. Growth of monolayer, discretely distributed, triangular single-crystal grains (with side lengths of tens to one hundred micrometers) on a sapphire substrate via chemical vapor deposition. A 3D piezo-nanostage (E545, Physik Instrumente), with nanoscale resolution, was employed to enable precise adjustments of the sample position during structure processing. The designed forked grating structure was fabricated using femtosecond laser direct writing for single-point removal processing. The femtosecond laser source is a mode-locked Ti: Sapphire ultrafast oscillator (Chameleon Vision-S, Coherent Inc.) with a central wavelength of 796 nm, a pulse width of 75 fs, and a repetition rate of 80 MHz, and focuses on the sample through a 100× air objective lens (NA = 0.95, Olympus) for patterning.

### Experimental setup for SH measurements

Optical measurements were performed using the identical femtosecond laser as employed for sample fabrication. The phase-only reflective liquid-crystal spatial light modulator (Pluto NIR-2, Holoeye Photonics AG) has 1920 × 1080 pixels, with a pixel pitch of 8 μm, on which computer-generated holograms with different topological charges can be displayed. The SHG signal is collected using a 50× air objective lens (NA = 0.8, Olympus). A low-pass filter (cut-off wavelength 500 nm, FESH0500, THORLABS) was installed in the optical path to filter out the fundamental frequency signal. The SH intensity distribution was caught by a charge-coupled device camera (acA4024-29uc, Basler camera, image area: 1024 × 1040 pixels) with the acquisition time of 100 ms. The pump power of the incident vortex beams was tunable for achieving clarified SH optical images on the CCD camera and the average pump power used for imaging was approximately 94.7 mW.

### Numerical simulation

The vortex beams were modeled using Laguerre-Gaussian (LG) modes. To account for the nonlinear effects, the local nonlinear polarization was assumed to be proportional to the intensity distribution, expressed as *P* ∝ ∣*E*∣^2^. The beam waist radius was set to 5 μm, consistent with the experimental parameters. The simulated fork grating possessed a spatial period of 800 nm. Based on scalar diffraction theory, we calculated the nonlinear diffraction patterns and subsequently employed the Fast Fourier Transform (FFT) algorithm to obtain the intensity distributions in the far-field *k*-space (spatial frequency domain).

To provide a clearer understanding of our numerical approach, we have expanded this section to detail the implementation of the scalar diffraction model. While this model focuses on the core mechanism of nonlinear directional emission, it effectively reproduces the experimental far-field patterns, with further vectorial analysis of the monolayer properties reserved for future study. Slight discrepancies in the simulation parameters were introduced to compensate for systematic errors present in the experimental setup.

## Supplementary Materials

### The PDF file includes:

Supplementary Text Figs. S1 to S8 Table S1 Legend for movie S1 References
