# Supplementary material for: Nonlinear directional emission on monolayer MoS2 by optical orbit-orbit coupling
Source: Sci Adv. 2026 Sep 30;12(40):eaei3860. doi: 10.1126/sciadv.aei3860 (PMC13626083; doi:10.1126/sciadv.aei3860)
Supplement: Supplementary file 1 [file sciadv.aei3860_sm.pdf]

Science Advances
AAAS

Supplementary Materials for

Nonlinear directional emission on monolayer MoS_2 by optical orbit-orbit coupling

Xianglong Wang *et al.*

Corresponding author: Yanlei Hu, huyl@ustc.edu.cn; Dong Wu, dongwu@ustc.edu.cn; Jincheng Ni, njc@ustc.edu.cn

Sci. Adv. **12**, eaei3860 (2026)
DOI: 10.1126/sciadv.aei3860

The PDF file includes:

Supplementary Text
Figs. S1 to S8
Table S1
Legend for movie S1
References

Other Supplementary Material for this manuscript includes the following:

Movie S1

Supplementary Text

Note 1: Angular momentum correlation mechanism in monolayer MoS_2.

Angular momentum locking is an interesting phenomenon in monolayer MoS_2 by nonlinear light-matter interaction. For example, the spin-angular-momentum locking can be realized in monolayer MoS_2. As widely reported, this phenomenon arises from the strong spin-orbit coupling and the broken inversion symmetry in the monolayer (*24–25*). Specifically, the valence band spin-splitting at the K and K' valleys (the two inequivalent corners of the Brillouin zone) is significant due to the strong spin-orbit interaction. This leads to a coupling between the spin and valley degrees of freedom.

Due to the broken inversion symmetry and the strong spin-orbit coupling, circularly polarized light selectively excites electrons in one of the valleys. Right-circularly polarized (σ^+) light excites electrons with spin up in the K valley, while left-circularly polarized (σ^-) light excites electrons with spin down in the K' valley. This selective excitation is the essence of the valley-selective optical pumping, which is a consequence of the spin-valley locking. Conversely, the valley and spin of the electron also determine the polarization of the emitted photon during recombination.

As reported in numerous publications, a nonlinear spin locking mechanism also exists in monolayer MoS_2 (*58*). In the process of light-matter interaction in two-dimensional materials, threefold rotational symmetry requires the conservation of total angular momentum. Therefore, the valley angular momentum $\Delta\tau\hbar$, exciton angular momentum $\Delta n\hbar$, lattice angular momentum $3N\hbar$, and photon spin angular momentum $\Delta m\hbar$ of the material will satisfy the following relationship:

$$\Delta m\hbar = \Delta\tau\hbar + \Delta n\hbar + 3N\hbar \tag{S1}$$

Thus, during two-photon absorption, the change in spin angular momentum will cause changes in valley, exciton, and lattice angular momentum. Based on this selection rule, two-photon processes such as two-photon fluorescence and second-harmonic generation will exhibit a valley-exciton locking effect. This means that under exciton resonance conditions, pure σ^- or σ^+ excitations will only occur in specific valleys (fig. S1A).

Conservation of orbital angular momentum also applies in two-photon absorption processes. It has been reported that Laguerre-Gaussian (LG) modes can undergo second-harmonic generation and that resulting in a frequency-doubled output beam that exhibits a higher-order mode (*42*). The LG mode with $p = 0$ can be expressed by

$$E^{(\omega)} = E_0 \exp\left(\frac{-r^2}{w^2}\right) \exp(-i\ell\phi) \left(\frac{r\sqrt{2}}{w}\right)^{\ell} \tag{S2}$$

where E^{ω} represents the complex electric field amplitude, E_0 is the peak electric field amplitude, r is the radial distance from the beam axis, ϕ is the azimuthal angle, w is the beam waist and ℓ is the azimuthal mode index, representing the number of helical twists in the wavefront.

Using a naive theory of SHG, the complex amplitude of the second harmonic field is proportional to the square of the complex amplitude of the fundamental field; that is,

$$E^{(2\omega)} \propto \left(E^{(w)}\right)^2 \tag{S3}$$

Combining this with Equation S2, we can deduce that the frequency-doubled beam retains a Laguerre-Gaussian mode with $p = 0$, but and the ℓ index has become 2ℓ and w is again reduced by $\sqrt{2}\ell$.

It follows that not only has the frequency been doubled, so has the orbital angular momentum per photon. Different from spin-valley locking where frequency doubling transitions of different spins

occur in different valleys, the doubling of orbital angular momentum in the SHG process can occur in any valley (fig. S1B).

Distinct from the flipping of the SAM that depends on the symmetry of the monolayer material, the doubling of the OAM can be attributed to the angular momentum conservation which is independent of the material symmetry.

Note 2: Theoretical derivation of directional second-harmonic emission based on monolayer MoS_2.

First, we define a linearly polarized Laguerre-Gaussian beam as:

$$E\ (r,\phi) = E_0 \exp\left(\frac{-r^2}{w^2}\right)\exp(-i\ell\phi)\left(\frac{r\sqrt{2}}{w}\right)^{\ell} \cdot \left(\cos(\vartheta)\, e_x + \sin(\vartheta)\, e_y\right) \qquad \text{(S4)}$$

where E represents the complex electric field amplitude, E_0 is the peak electric field amplitude, r is the radial distance from the beam axis, ϕ is the azimuthal angle, w is the beam waist and ℓ is the azimuthal mode index, representing the number of helical twists in the wavefront. The ϑ defines angle between the armchair axis of the crystal and the polarization-axis of the fundamental beam and $x(y)$ refers to armchair (zig-zag) axis of the crystal.

It has been reported that monolayer MoS_2 belongs to the D_{3h} point group only at the Γ point, whereas it belongs to the C_{3h} point group at the ±K valleys near the resonance frequency (*29*). The second-order nonlinear susceptibility $\chi^{(2)}$ of the C_{3h} point group can be described as: $\chi^{(2)}_{xxx} = -\chi^{(2)}_{xyy} = -\chi^{(2)}_{yyx} = -\chi^{(2)}_{yxy} = \chi^{(2)}_0$ and $\chi^{(2)}_{yyy} = -\chi^{(2)}_{yxx} = -\chi^{(2)}_{xxy} = -\chi^{(2)}_{xyx}$. $\chi^{(2)}_{yyy}$ contributions from both ±K valleys, $\chi^{(2)}_{yyy} = \chi^{(2)}_{yyy}(\mathrm{K}) + \chi^{(2)}_{yyy}(-\mathrm{K})$. Since the ±K valleys are connected by time-reversal symmetry (TRS), then $\chi^{(2)}_{yyy}(\mathrm{K}) = -\chi^{(2)}_{yyy}(-\mathrm{K})$. In this condition, the second-order polarization is given by:

$$\begin{pmatrix} P_x^{(2)} \\ P_y^{(2)} \end{pmatrix} = \epsilon_0 \sum\nolimits_{\tau=\pm\mathrm{K}} \begin{pmatrix} \chi^{(2)}_{xxx}(\tau) & -\chi^{(2)}_{xxx}(\tau) & -\chi^{(2)}_{yyy}(\tau) \\ -\chi^{(2)}_{yyy}(\tau) & \chi^{(2)}_{yyy}(\tau) & -\chi^{(2)}_{xxx}(\tau) \end{pmatrix} \cdot \begin{pmatrix} E_x^2 \\ E_y^2 \\ 2E_xE_y \end{pmatrix}$$

$$= \epsilon_0 \begin{pmatrix} \chi^{(2)}_{xxx}\left(E_x^2 - E_y^2\right) - 2\tau\chi^{(2)}_{yyy}E_xE_y \\ -2\chi^{(2)}_{xxx}E_xE_y - \tau\chi^{(2)}_{yyy}\left(E_x^2 - E_y^2\right) \end{pmatrix} \qquad \text{(S5)}$$

Note the valley index $\tau = \pm 1$ is equivalent to ±K in front of the $\chi^{(2)}_{yyy}$ term.

Under linear polarization, $\chi^{(2)}_{yyy} = \chi^{(2)}_{yyy}(\mathrm{K}) + \chi^{(2)}_{yyy}(-\mathrm{K}) = 0$, $\chi^{(2)}_{yyy}$ is disappears. Substituting Eq. (S4) into Eq. (S5) yields:

$$\begin{pmatrix} P_x^{(2)} \\ P_y^{(2)} \end{pmatrix} = \begin{pmatrix} \epsilon_0\chi_0^{(2)}\left(E_x^2 - E_y^2\right) \\ -2\epsilon_0\chi_0^{(2)}E_xE_y \end{pmatrix}$$

$$= \begin{pmatrix} \epsilon_0\chi_0^{(2)}E_0^2 \exp\left(\dfrac{-2r^2}{w^2}\right)\exp(-i\cdot 2\ell\phi)\left(\dfrac{r\sqrt{2}}{w}\right)^{2\ell} \cdot \cos(2\vartheta) \\ -\epsilon_0\chi_0^{(2)}E_0^2 \exp\left(\dfrac{-2r^2}{w^2}\right)\exp(-i\cdot 2\ell\phi)\left(\dfrac{r\sqrt{2}}{w}\right)^{2\ell} \cdot \sin(2\vartheta) \end{pmatrix}$$

Consequently, we get

$$P^{(2)}\ (r,\phi) = \epsilon_0\chi_0^{(2)}E_0^2 \exp\left(\frac{-2r^2}{w^2}\right)\exp(-i\cdot 2\ell\phi)\left(\frac{r\sqrt{2}}{w}\right)^{2\ell} \cdot \left(\cos(-2\vartheta)\, e_x + \sin(-2\vartheta)\, e_y\right) \qquad \text{(S6)}$$

According to Eq. (S3), we can deduce that the frequency-doubled beam retains a Laguerre-Gaussian mode, and the ℓ index has become 2ℓ and w is again reduced by $\sqrt{2}\ell$. In addition, the

polarization direction of the beam changes, with the angle relative to the x-axis shifting from θ to -2θ.
In the case of the patterned fork grating structure, the expression is given by:

$$\chi_{\text{eff}}^{(2)}(r,\phi) = \frac{1}{2} + \frac{1}{2}\text{sign}\left[\cos\left\{m\phi + \frac{2\pi}{\Lambda}(\cos(\theta)\,e_x + \sin(\theta)\,e_y)\right\}\right] \quad \text{(S7)}$$

where m is the mode of the structure, ϕ is the azimuthal angle, $x(y)$ refers to armchair (zig-zag) axis of the crystal and θ defines angle between the armchair axis of the crystal and the grating direction. The patterned fork grating structure imposes a spatial distribution on the second-order nonlinear susceptibility. By treating $m\phi$ as a perturbation of a periodic function, the nonlinear coefficient $\chi_{\text{eff}}^{(2)}(x,y)$can be expanded into a Fourier series as:

$$\chi_{\text{eff}}^{(2)}(r,\phi) \approx \chi^{(2)}\sum_k a_m \exp\left[ik(m\phi + \frac{2\pi}{\Lambda}(\cos(\theta)\,e_x + \sin(\theta)\,e_y))\right]\exp(\tfrac{1}{2}i) \quad \text{(S8)}$$

In Eq. (S5), a_m is the amplitude of the kth-order Fourier component. When the incident beam carries an optical vortex, from Eqs. (S4–S8), we have

$$P^{(2)}(r,\phi) = \epsilon_0\chi_0^{(2)}\sum_k a_m \exp\left[ik\left(m\phi + \frac{2\pi}{\Lambda}\left(\cos(\theta)\,e_x + \sin(\theta)\,e_y\right)\right)\right]\exp\left(\frac{1}{2}\,i\right)\cdot E_0^2\exp\left(\frac{-2r^2}{w^2}\right)\exp(-i\cdot 2\ell\phi)\left(\frac{r\sqrt{2}}{w}\right)^{2\ell}\cdot\left(\cos(-2\vartheta)\,e_x + \sin(-2\vartheta)\,e_y\right)$$

$$P^{(2)}(r,\phi) = \epsilon_0\chi_0^{(2)}\sum_k a_m E_0^2\left(\frac{r\sqrt{2}}{w}\right)^{2\ell}\cdot\exp\left(\frac{-2r^2}{w^2}\right)\exp\left(\frac{1}{2}i\right)\exp\left[i((2\ell + km)\phi + \frac{2\pi k}{\Lambda}\left(\cos(\theta)\,e_x + \sin(\theta)\,e_y\right)\right]\cdot\left(\cos(-2\vartheta)\,e_x + \sin(-2\vartheta)\,e_y\right) \quad \text{(S9)}$$

In Eq. (S9), we can see that the kth diffraction order is still a beam carrying a spiral phase, with a topological charge of $2\ell + km$.

.

Note 3: The influence of circular polarization valley selection rules in monolayer MoS_2 on experimental results.

Here, we consider vortex beams carrying spin angular momentum.

$$E\,(r,\phi) = E_0\exp\left(\frac{-r^2}{w^2}\right)\exp(-i\ell\phi)\left(\frac{r\sqrt{2}}{w}\right)^{\ell}\sigma_+ \quad \text{(S10)}$$

where σ_+ describes the circular polarization state of the beam. When we transform from the linear polarization basis to the circular polarization basis, we have

$$E_\pm = \frac{1}{\sqrt{2}}(E_x \pm iE_y) \quad \text{(S11)}$$

$$P_\pm = \frac{1}{\sqrt{2}}(P_x \pm iP_y) \quad \text{(S12)}$$

from Eq. (S2),

$$P^{(2)} = \begin{pmatrix}P_+\\P_-\end{pmatrix} = \frac{1}{\sqrt{2}}\epsilon_0\begin{pmatrix}\chi_{xxx}^{(2)}\left(E_x^2 - E_y^2 - 2iE_xE_y\right) - i\tau\chi_{yyy}^{(2)}\left(E_x^2 - E_y^2 - 2iE_xE_y\right)\\ \chi_{xxx}^{(2)}\left(E_x^2 - E_y^2 + 2iE_xE_y\right) - i\tau\chi_{yyy}^{(2)}\left(E_x^2 - E_y^2 + 2iE_xE_y\right)\end{pmatrix} = \epsilon_0\begin{pmatrix}\left(\chi_{xxx}^{(2)} - i\tau\chi_{yyy}^{(2)}\right)E_-^2\\ \left(\chi_{xxx}^{(2)} + i\tau\chi_{yyy}^{(2)}\right)E_+^2\end{pmatrix} \quad \text{(S13)}$$

From Eq. (S10), when the incident OAM beam carries a SAM of σ_+,

$$P^{(2)} = \epsilon_0\left(\chi_{xxx}^{(2)} - i\tau\chi_{yyy}^{(2)}\right)E_0^2\exp\left(\frac{-2r^2}{w^2}\right)\exp(-i\cdot 2\ell\phi)\left(\frac{r\sqrt{2}}{w}\right)^{2\ell}\sigma_- \quad \text{(S14)}$$

And when the incident OAM beam carries a SAM of σ_-,

$$P^{(2)} = \epsilon_0\left(\chi_{xxx}^{(2)} - i\tau\chi_{yyy}^{(2)}\right)E_0^2\exp\left(\frac{-2r^2}{w^2}\right)\exp(-i\cdot 2\ell\phi)\left(\frac{r\sqrt{2}}{w}\right)^{2\ell}\sigma_+ \quad \text{(S15)}$$

However, the variation of the total angular momentum differs slightly in SHG with vortex beam configurations, since the process involves a doubling of the topological charge. Consequently, the total angular momentum satisfies:

$$J_{in} = \ell + \sigma \text{ and } J_{out} = 2\ell - \sigma \tag{S16}$$

In general, within the configuration of structured light beams, the valley selection rules for monolayer MoS_2 remain valid, primarily influencing the local polarization characteristics. Regarding nonlinear selection rules, the helicity of the SHG is opposite to that of the fundamental beam.

Note 4: Simulated realization of multi-dimensional directional SH emission.

To explore the SH directional emission of higher dimensional fork gratings, we performed relevant simulation analysis (fig. S8). We generated schematic diagrams of 1D, 2D, 3D, and 4D fork gratings, respectively (figs. S8A–D). The dimensionality of the grating is defined as N. Specifically, the angle between the N-dimensional grating is $\theta = \pi/N$. And the fork gratings were incident by OAM with $\ell = 3, 4, 8, 8$, respectively. Figures S8E–H illustrate the *k*-space directional SH emission from fork gratings with different dimensions. As we can see, the area of the patterned structure decreases with increasing of N. We have experimentally demonstrated that the ratio of SHG intensity from 1D patterned structure to original material is A ($\approx$ 48%), which is related to the remaining patterned area. And the area ratio of an N-dimensional patterned structure to original material is given by $\eta_1 \approx A^N$ (fig. S8I). The relationship between the simulated diffraction efficiency η_2 and the structural dimension N is shown in fig. S8J. And the dependence of total efficiency ($\eta = \eta_1 \times \eta_2$) on dimensionality is shown in fig. S8K. The efficiency decreases with increasing dimension N, then stabilizes for $N > 6$. Theoretically, the emission dimension can be infinite. However, in practical applications, limited by factors such as fabrication precision, the maximum achievable dimension with this method is approximately 5 to 6. However, alternative approaches can be employed to address the issue of low efficiency, for example, by fabricating gratings in different regions.

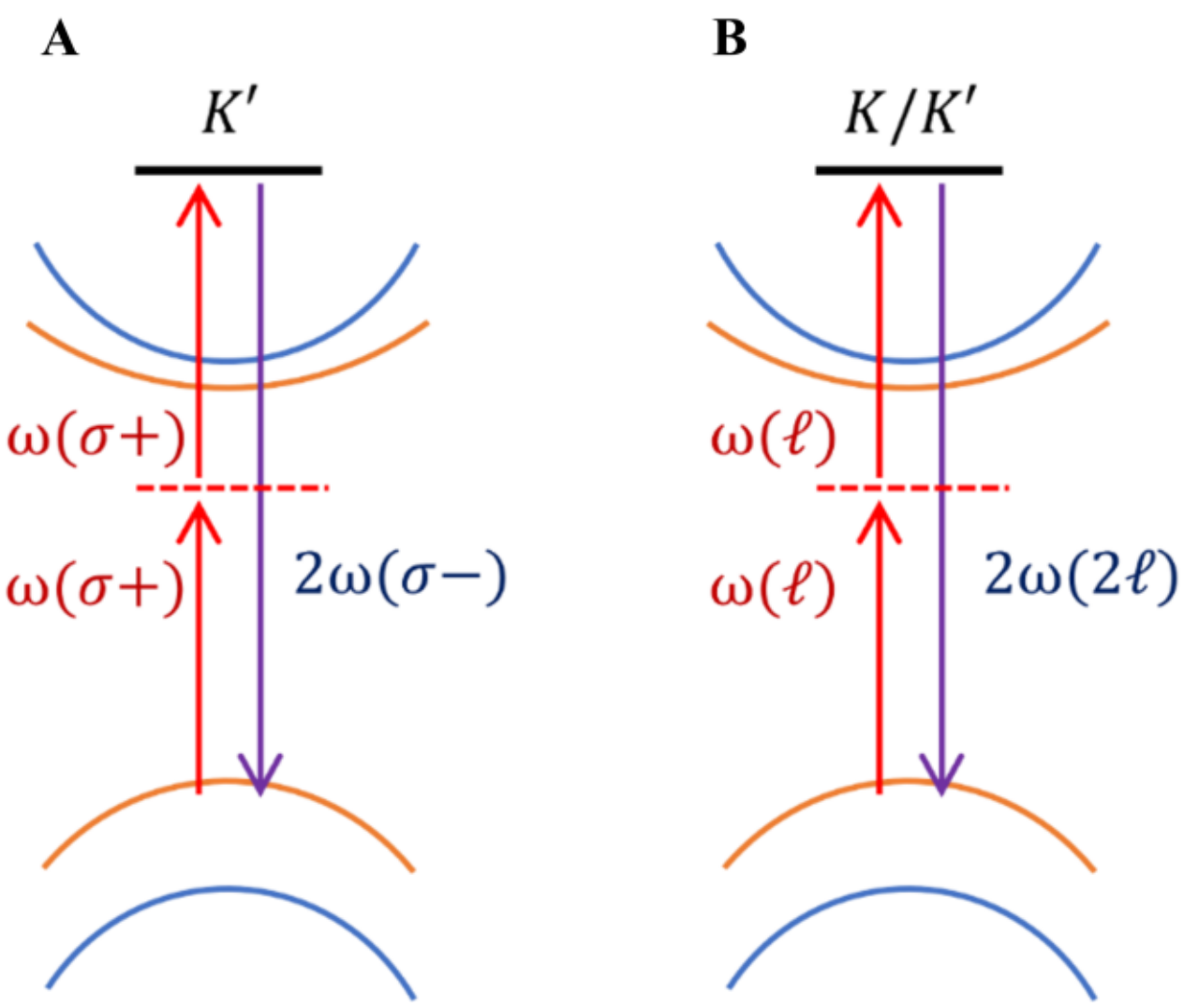

Fig. S1. The angular momentum locking mechanism in SHG of monolayer MoS_2. (**A**) Spin-valley locking mechanism. (**B**) Orbital angular momentum conservation mechanism.

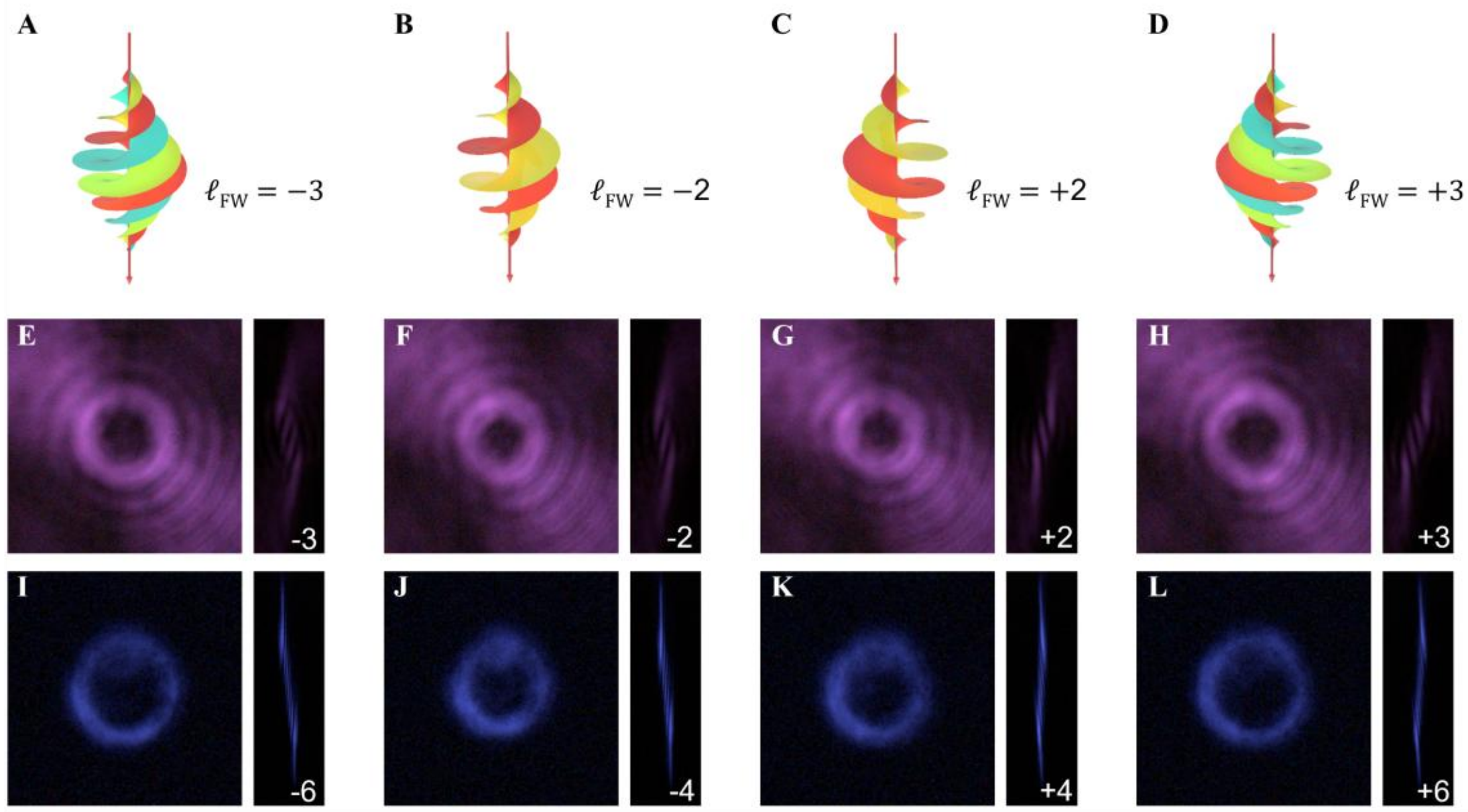

Fig. S2. Experimentally measured topological charges of the fundamental and second-harmonic vortex beams. (**A** to **D**) correspond to topological charges of $\boldsymbol{\ell} = -\mathbf{3}$, $-\mathbf{2}$, $+\mathbf{2}$, and $+\mathbf{3}$, respectively. (**E** to **H**) Measured intensity profiles (left) and cylindrical-lens-converted lobe patterns (right) for vortex beams with topological charges of $\boldsymbol{\ell} = -\mathbf{3}, -\mathbf{2}, +\mathbf{2}$, and $+\mathbf{3}$. (**I** to **L**) Measured SH intensity profiles (left) and cylindrical-lens-converted lobe patterns (right).

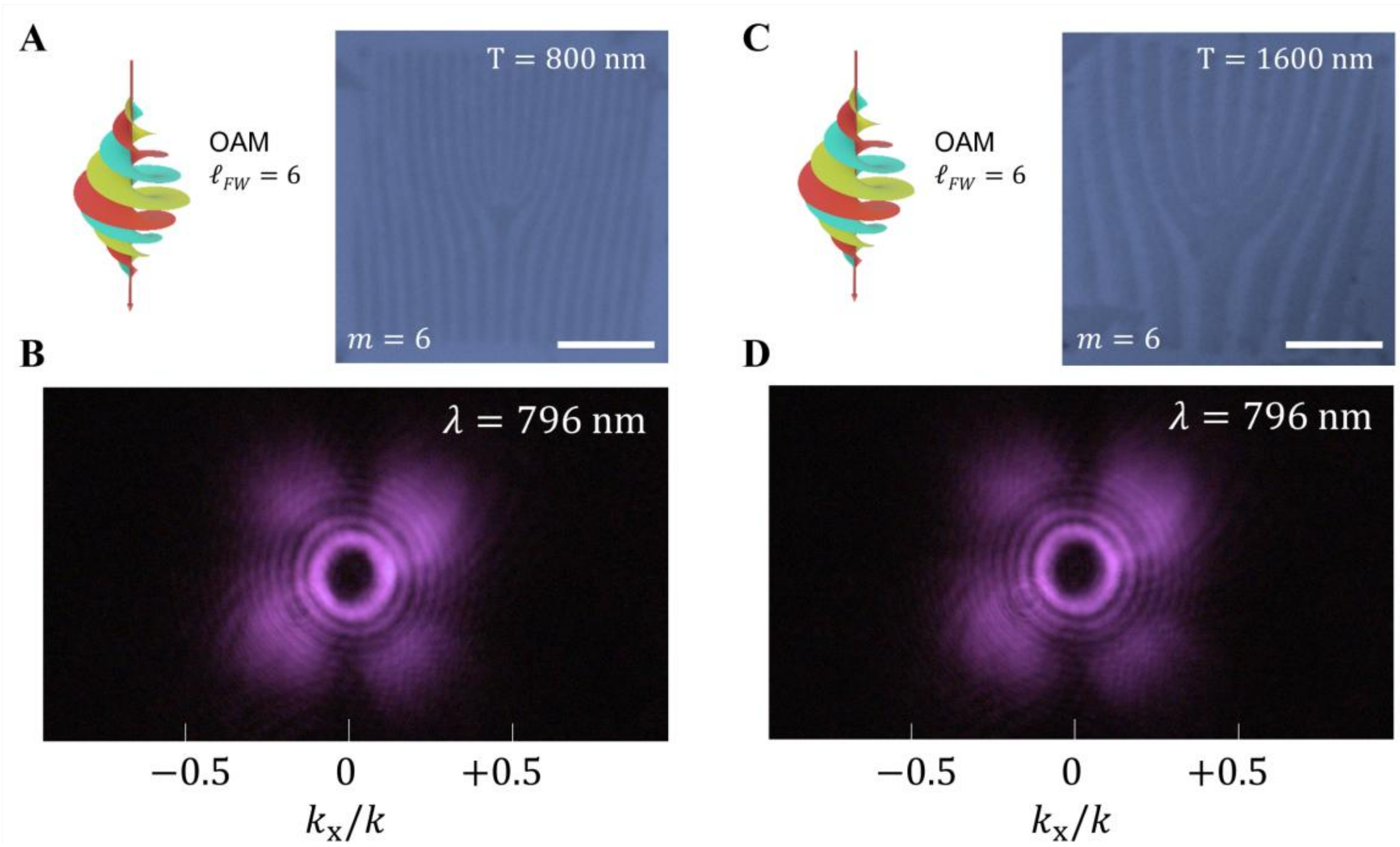

Fig. S3. Linear response of patterned monolayer MoS_2. (**A**) Schematic illustration of an incident OAM beam with $\boldsymbol{\ell = 6}$ (left). Optical micrograph (adapted from Figure 3D with 167% magnification, 0° rotation, and no flipping) of the $\boldsymbol{m = 6, \mathrm{T} = 800\ \mathrm{nm}}$ fork nanograting (right). (**B**) Experimental linear *k*-space imaging under the optical configuration of (A). (**C**) Schematic illustration of an incident OAM beam with $\boldsymbol{\ell = 6}$ (left) and optical micrograph of the $\boldsymbol{m = 6, \mathrm{T} = 1600\ \mathrm{nm}}$ fork nanograting (right). (**D**) Experimental linear *k*-space imaging under the optical configuration of (C).

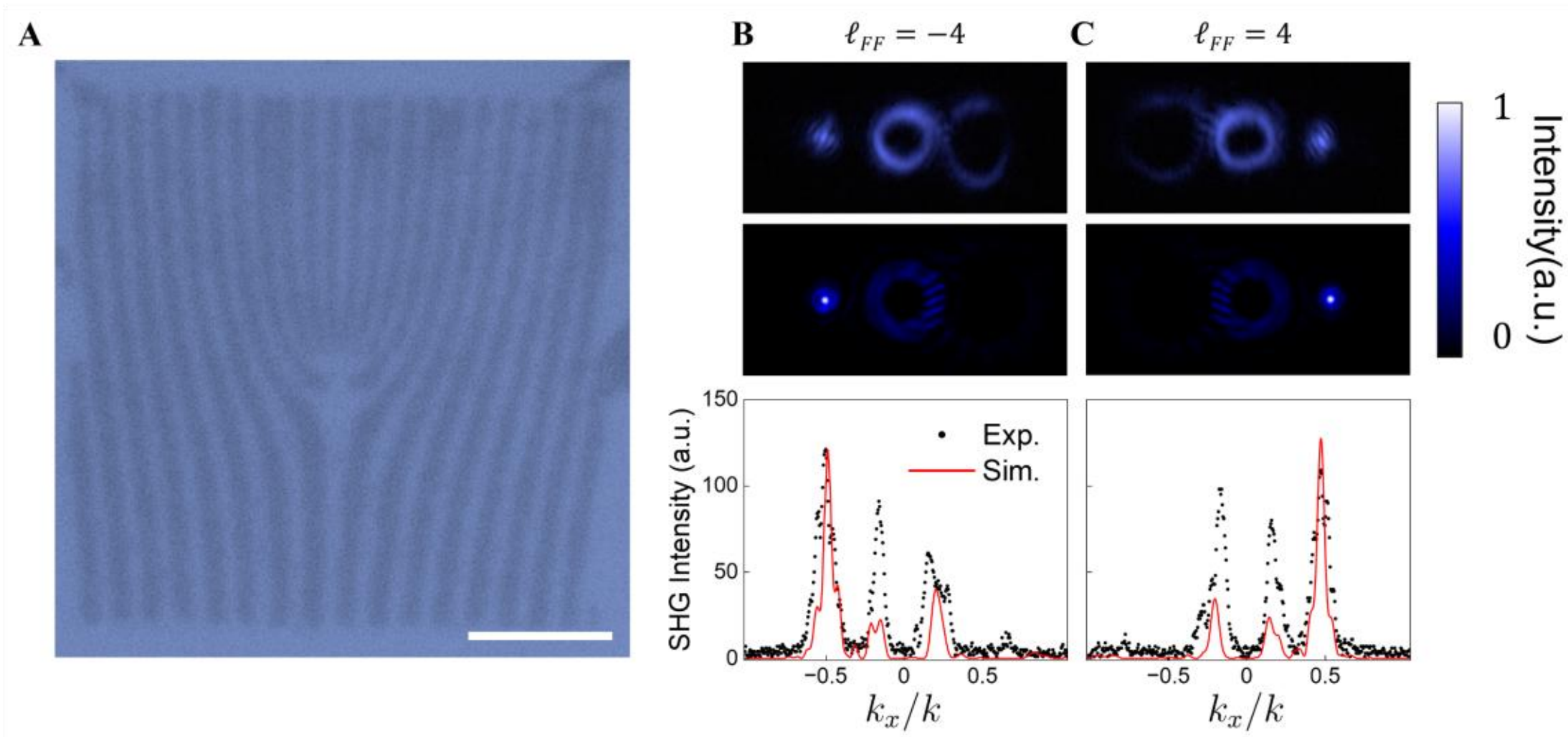

Fig. S4. Directional emission with $m = 8$. (**A**) Optical image of the grating structure. Scale bar, 5μm. (**B** and **C**) Experimental (top panel) and simulated (middle panel) SH far-field k-space imaging and line cut of the intensity profiles (bottom panel) along the fork grating system excited by the vortex beam with $\boldsymbol{\ell}_{\mathbf{FW}} = \mathbf{-4}$ (B) and $\mathbf{+4}$ (C), respectively.

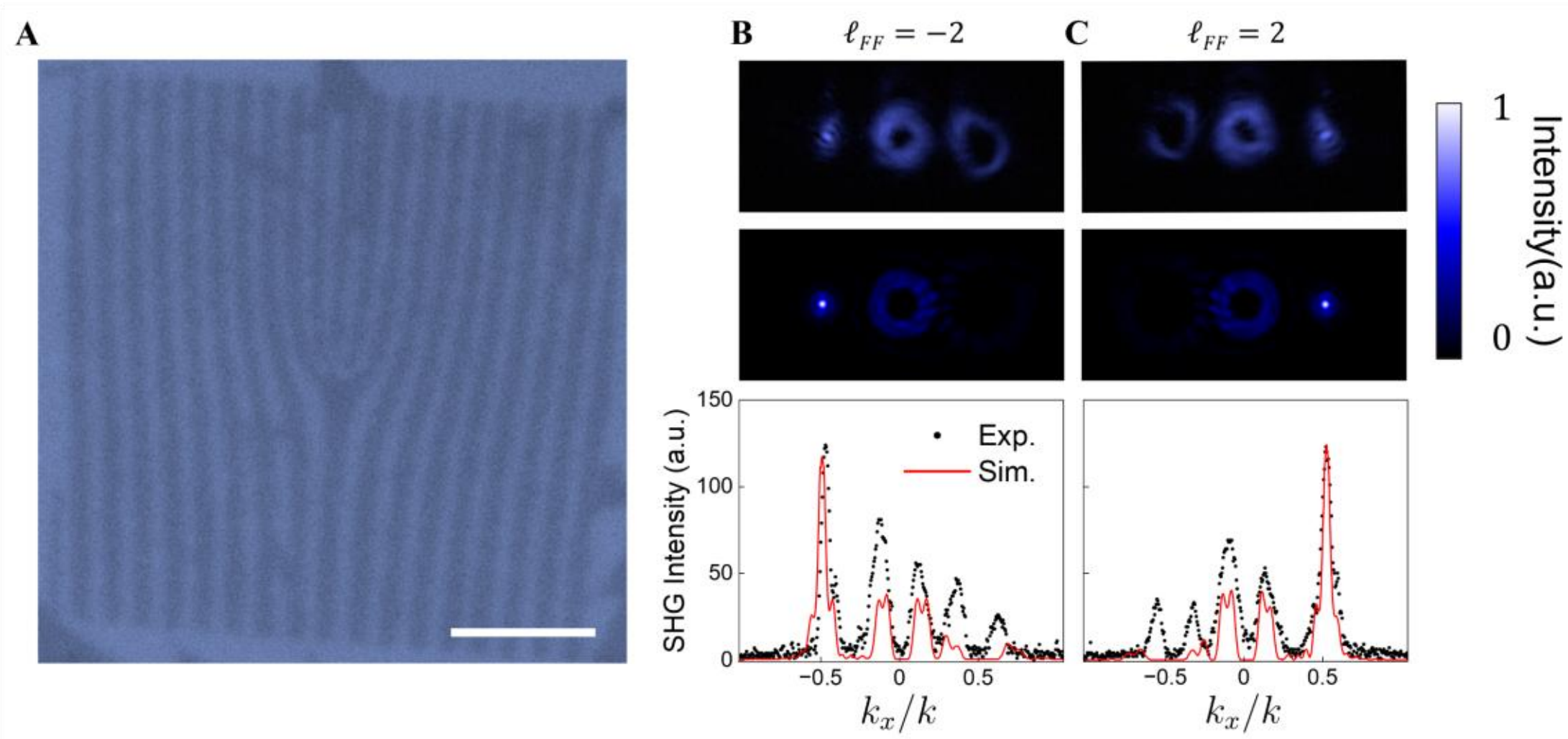

Fig. S5. Directional emission with $m = 4$. (**A**) Optical image of the grating structure. Scale bar, 5μm. (**B** and **C**) Experimental (top panel) and simulated (middle panel) SH far-field k-space imaging and line cut of the intensity profiles (bottom panel) along the fork grating system excited by the vortex beam with $\boldsymbol{\ell}_{\mathbf{FW}} = -\mathbf{2}$ (B) and $+\mathbf{2}$ (C), respectively.

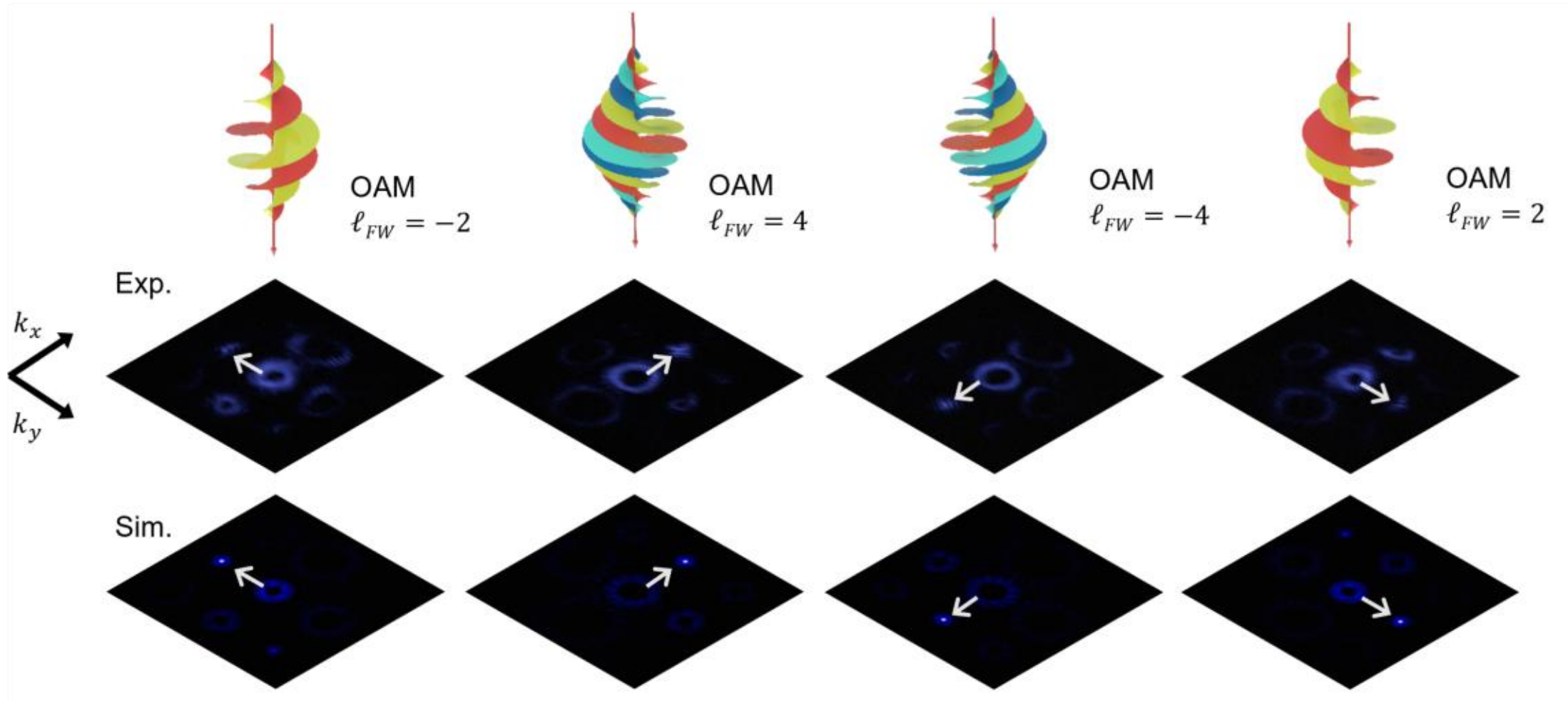

Fig. S6. Directional emission with $m_x = 8, m_y = 4$. Schematic diagram (top) of vortex beams with topological charges of -2, 4, -4, and 2, along with simulated (middle) and experimental (bottom) *k*-space images.

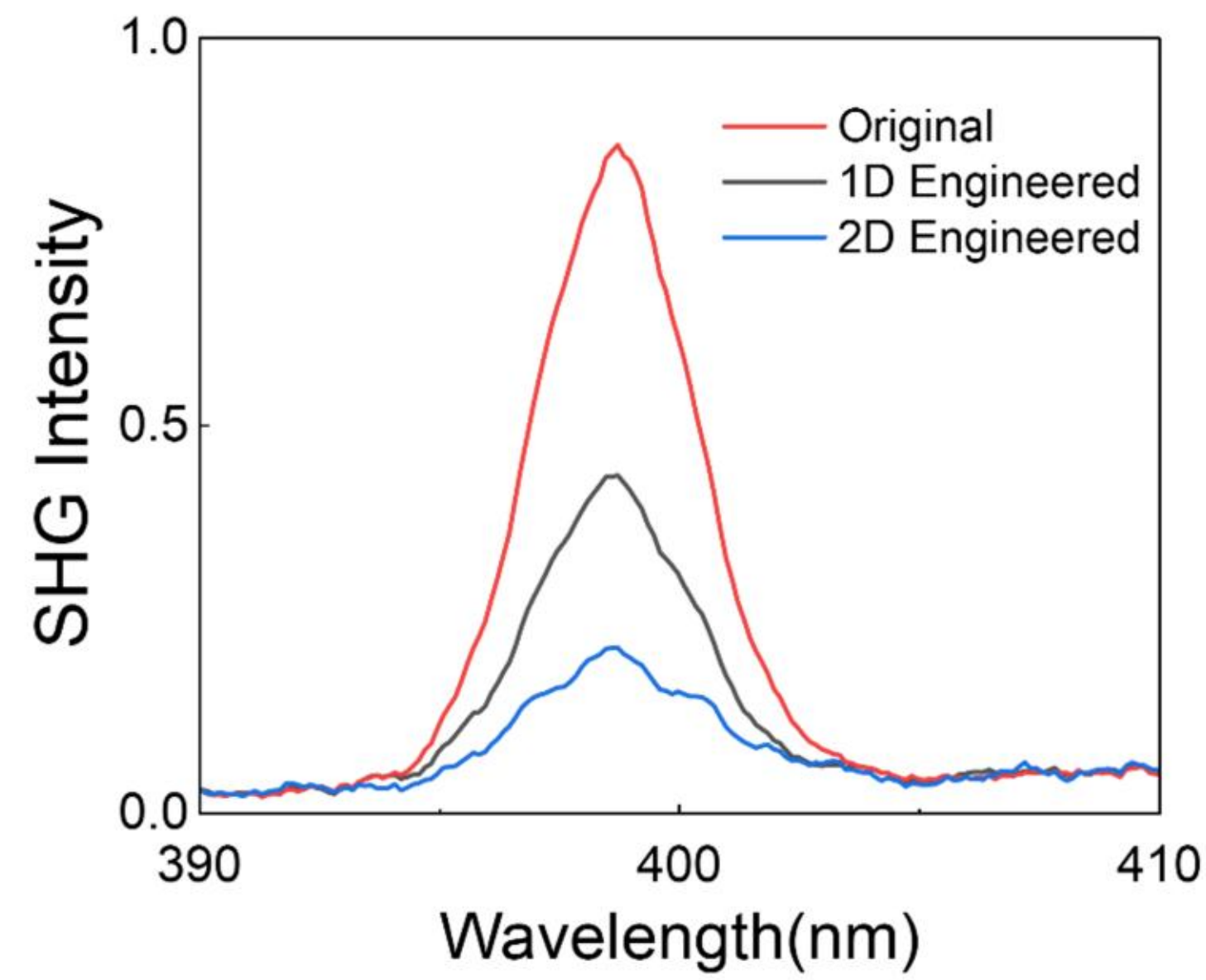

Fig. S7. SHG spectra excited on monolayer MoS_2 on the original MoS_2, the 1D fork grating, and the 2D grating structure, respectively.

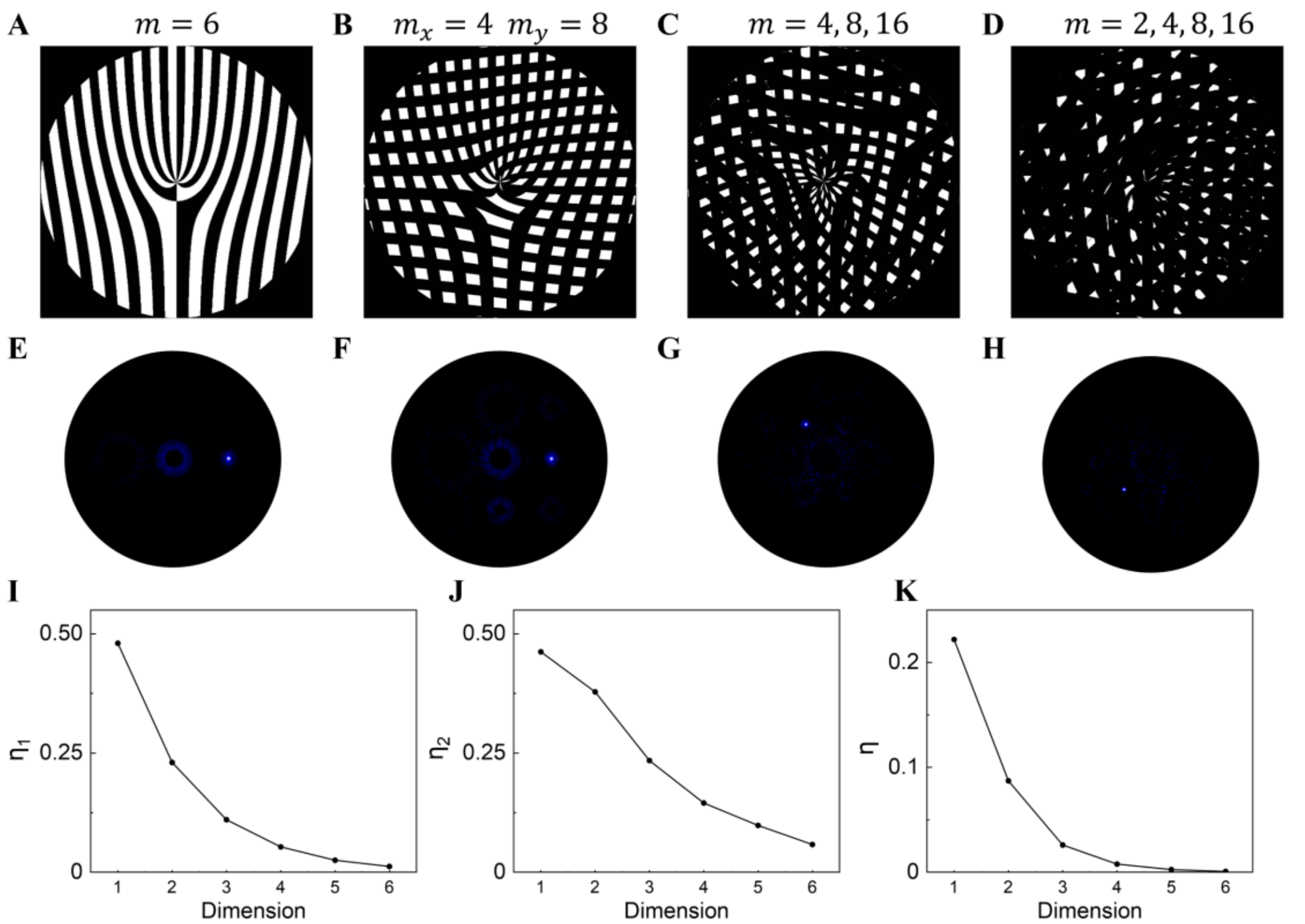

Fig. S8. Simulation of multidimensional directional emission of SHG. (**A** to **D**) Schematic diagrams of 1D (A), 2D (B), 3D (C), and 4D (D) fork gratings, respectively. (**E** to **H**) Simulation results of *k*-space SHG emission from fork gratings with different dimensionalities. (**I**) Dependence of the ratio ($\boldsymbol{\eta_1}$) of patterned residual area to total area on dimensionality. (**J**) Dependence of simulated diffraction efficiency ($\boldsymbol{\eta_2}$) on fork grating dimensionality. (**K**) Dependence of total efficiency ($\boldsymbol{\eta} = \boldsymbol{\eta_1} \times \boldsymbol{\eta_2}$) on dimensionality.

Table S1. Comparisons of the directionality by devices with different thicknesses

Reference	Angular momentum	Device	Thickness	Operating wavelength	Directionality	Number of states
3	SAM	metasurface	520nm	1500nm	0.95	2
4	SAM	photonic crystal	120nm	594nm	0.4	2
59	SAM	photonic crystal	400nm	458nm	0.33	2
5	OAM	waveguide	3.8μm	800nm	0.59	2
6	SAM	waveguide	~185nm	810nm	0.44	2
7	SAM	waveguide	~500nm	594nm	0.35	2
19	OAM	waveguide	750nm	1064nm	0.82	2
**Our work**	**OAM**	**monolayer patterned**	**~0.7nm**	**796nm**	**0.84**	**2/4**

Movie S1. The encoding, transmission, and decoding of the characters “OV”.

REFERENCES

1. A. G. Curto, G. Volpe, T. H. Taminiau, M. P. Kreuzer, R. Quidant, N. F. van Hulst, Unidirectional emission of a quantum dot coupled to a nanoantenna. *Science* **329**, 930–933 (2010).

2. C. Dai, S. Wan, Z. Li, Y. Shi, S. Zhang, Z. Li, Switchable unidirectional emissions from hydrogel gratings with integrated carbon quantum dots. *Nat. Commun.* **15**, 845 (2024).

3. A. Sinelnik, S. H. Lam, F. Coviello, S. Klimmer, G. Della Valle, D.-Y. Choi, T. Pertsch, G. Soavi, I. Staude, Ultrafast all-optical second harmonic wavefront shaping. *Nat. Commun.* **15**, 2507 (2024).

4. X. Duan, B. Wang, K. Rong, C. Liu, V. Gorovoy, S. Mukherjee, V. Kleiner, E. Koren, E. Hasman, Valley-addressable monolayer lasing through spin-controlled Berry phase photonic cavities. *Science* **381**, 1429–1432 (2023).

5. J. Ni, S. Ji, Z. Wang, S. Liu, Y. Hu, Y. Chen, J. Li, X. Li, J. Chu, D. Wu, C.-W. Qiu, Unidirectional unpolarized luminescence emission via vortex excitation. *Nat. Photonics* **17**, 601–606 (2023).

6. Y. Chen, S. Qian, K. Wang, X. Xing, A. Wee, K. P. Loh, B. Wang, D. Wu, J. Chu, A. Alù, P. Lu, C.-W. Qiu, Chirality-dependent unidirectional routing of WS_2 valley photons in a nanocircuit. *Nat. Nanotechnol.* **17**, 1178–1182 (2022).

7. S.-H. Gong, F. Alpeggiani, B. Sciacca, E. C. Garnett, L. Kuipers, Nanoscale chiral valley-photon interface through optical spin-orbit coupling. *Science* **359**, 443–447 (2018).

8. R. Shreiner, K. Hao, A. Butcher, A. A. High, Electrically controllable chirality in a nanophotonic interface with a two-dimensional semiconductor. *Nat. Photonics* **16**, 330–336 (2022).

9. P.-G. Chen, Z. Li, Y. Qi, T. W. Lo, S. Wang, W. Jin, K.-Y. Wong, S. Fan, A. V. Zayats, D. Lei, Long-range directional routing and spatial selection of high-spin-purity valley trion emission in monolayer WS_2. *ACS Nano* **15**, 18163–18171 (2021).

10. Y. Chen, J. Feng, Y. Huang, W. Chen, R. Su, S. Ghosh, Y. Hou, Q. Xiong, C.-W. Qiu, Compact spin-valley-locked perovskite emission. *Nat. Mater.* **22**, 1065–1070 (2023).

11. G. Hu, X. Hong, K. Wang, J. Wu, H.-X. Xu, W. Zhao, W. Liu, S. Zhang, F. Garcia-Vidal, B. Wang, P. Lu, C.-W. Qiu, Coherent steering of nonlinear chiral valley photons with a synthetic Au–WS_2 metasurface. *Nat. Photonics* **13**, 467–472 (2019).

12. M. Li, C. U. Hail, S. Biswas, H. A. Atwater, Excitonic beam steering in an active van der Waals metasurface. *Nano Lett.* **23**, 2771–2777 (2023).

13. K. Rong, B. Wang, A. Reuven, E. Maguid, B. Cohn, V. Kleiner, S. Katznelson, E. Koren, E. Hasman, Photonic Rashba effect from quantum emitters mediated by a Berry-phase defective photonic crystal. *Nat. Nanotechnol.* **15**, 927–933 (2020).

14. J. Wang, H. Li, Y. Ma, M. Zhao, W. Liu, B. Wang, S. Wu, X. Liu, L. Shi, T. Jiang, J. Zi, Routing valley exciton emission of a WS_2 monolayer via delocalized Bloch modes of in-plane inversion-symmetry-broken photonic crystal slabs. *Light Sci. Appl.* **9**, 148 (2020).

15. M. Li, Q. Li, M. L. Brongersma, H. A. Atwater, Optical devices as thin as atoms. *Science* **386**, 1226–1228 (2024).

16. S. Klimmer, O. Ghaebi, Z. Gan, A. George, A. Turchanin, G. Cerullo, G. Soavi, All-optical polarization and amplitude modulation of second-harmonic generation in atomically thin semiconductors. *Nat. Photonics* **15**, 837–842 (2021).

17. A. Di Francescantonio, A. Zilli, D. Rocco, V. Vinel, L. Coudrat, F. Conti, P. Biagioni, L. Duò, A. Lemaître, C. De Angelis, G. Leo, M. Finazzi, M. Celebrano, All-optical free-space routing of upconverted light by metasurfaces via nonlinear interferometry. *Nat. Nanotechnol.* **19**, 298–305 (2024).

18. F. Tagarelli, E. Lopriore, D. Erkensten, R. Perea-Causín, S. Brem, J. Hagel, Z. Sun, G. Pasquale, K. Watanabe, T. Taniguchi, E. Malic, A. Kis, Electrical control of hybrid exciton transport in a van der Waals heterostructure. *Nat. Photonics* **17**, 615–621 (2023).

19. H. Lin, G. Zhang, G. Xue, W. Xie, W. Zhu, K. Liu, Z. Pan, Q. Gong, G. Lyu, Steering nonlinear chiral valley photons through optical orbit–orbit coupling. *Laser Photonics Rev.* **19**, 2400545 (2025).

20. L. Tang, X. Meng, D. Deng, X. Bao, Confinement catalysis with 2D materials for energy conversion. *Adv. Mater.* **31**, e1901996 (2019).

21. W. Zhang, K. Uwakwe, J. Hu, Y. Wei, J. Zhu, W. Zhou, C. Ma, L. Yu, R. Huang, D. Deng, Ambient-condition acetylene hydrogenation to ethylene over WS_2-confined atomic Pd sites. *Nat. Commun.* **15**, 9457 (2024).

22. C. Rong, T. Su, Z. Li, T. Chu, M. Zhu, Y. Yan, B. Zhang, F.-Z. Xuan, Elastic properties and tensile strength of 2D $Ti_3C_2T_x$ MXene monolayers. *Nat. Commun.* **15**, 1566 (2024).

23. S. Du, Y. Guo, X. Huang, C. Sun, Z. Zhang, L. Hu, R. Zheng, Q. Bai, A. Jin, H. Yang, Y. Zhang, J. Li, C. Gu, Strain lithography for two-dimensional materials by electron irradiation. *Appl. Phys. Lett.* **120**, 093104 (2022).

24. K. F. Mak, K. He, J. Shan, T. F. Heinz, Control of valley polarization in monolayer MoS_2 by optical helicity. *Nat. Nanotechnol.* **7**, 494–498 (2012).

25. H. Zeng, J. Dai, W. Yao, D. Xiao, X. Cui, Valley polarization in MoS_2 monolayers by optical pumping. *Nat. Nanotechnol.* **7**, 490–493 (2012).

26. G. Wang, A. Chernikov, M. M. Glazov, T. F. Heinz, X. Marie, T. Amand, B. Urbaszek, Colloquium: Excitons in atomically thin transition metal dichalcogenides. *Rev. Mod. Phys.* **90**, 021001 (2018).

27. H. Lin, Z.-Q. Xu, G. Cao, Y. Zhang, J. Zhou, Z. Wang, Z. Wan, Z. Liu, K. P. Loh, C.-W. Qiu, Q. Bao, B. Jia, Diffraction-limited imaging with monolayer 2D material-based ultrathin flat lenses. *Light Sci. Appl.* **9**, 137 (2020).

28. J. van de Groep, J.-H. Song, U. Celano, Q. Li, P. G. Kik, M. L. Brongersma, Exciton resonance tuning of an atomically thin lens. *Nat. Photonics* **14**, 426–430 (2020).

29. P. Herrmann, S. Klimmer, T. Lettau, T. Weickhardt, A. Papavasileiou, K. Mosina, Z. Sofer, I. Paradisanos, D. Kartashov, J. Wilhelm, G. Soavi, Nonlinear valley selection rules and all-optical probe of broken time-reversal symmetry in monolayer WSe_2. *Nat. Photonics* **19**, 300–306 (2025).

30. T. I. Andersen, R. J. Gelly, G. Scuri, B. L. Dwyer, D. S. Wild, R. Bekenstein, A. Sushko, J. Sung, Y. Zhou, A. A. Zibrov, X. Liu, A. Y. Joe, K. Watanabe, T. Taniguchi, S. F. Yelin, P. Kim, H. Park, M. D. Lukin, Beam steering at the nanosecond time scale with an atomically thin reflector. *Nat. Commun.* **13**, 3431 (2022).

31. C. Janisch, Y. Wang, D. Ma, N. Mehta, A. L. Elías, N. Perea-López, M. Terrones, V. Crespi, Z. Liu, Extraordinary second harmonic generation in tungsten disulfide monolayers. *Sci. Rep.* **4**, 5530 (2014).

32. L. Allen, M. W. Beijersbergen, R. J. C. Spreeuw, J. P. Woerdman, Orbital angular momentum of light and the transformation of Laguerre-Gaussian laser modes. *Phys. Rev. A* **45**, 8185–8189 (1992).

33. J. Ni, S. Liu, D. Wu, Z. Lao, Z. Wang, K. Huang, S. Ji, J. Li, Z. Huang, Q. Xiong, Y. Hu, J. Chu, C.-W. Qiu, Gigantic vortical differential scattering as a monochromatic probe for multiscale chiral structures. *Proc. Natl. Acad. Sci. U.S.A.* **118**, e2020055118 (2021).

34. X. Ouyang, Y. Xu, M. Xian, Z. Feng, L. Zhu, Y. Cao, S. Lan, B.-O. Guan, C.-W. Qiu, M. Gu, X. Li, Synthetic helical dichroism for six-dimensional optical orbital angular momentum multiplexing. *Nat. Photonics* **15**, 901–907 (2021).

35. P. Huo, W. Chen, Z. Zhang, Y. Zhang, M. Liu, P. Lin, H. Zhang, Z. Chen, H. Lezec, W. Zhu, A. Agrawal, C. Peng, Y. Lu, T. Xu, Observation of spatiotemporal optical vortices enabled by symmetry-breaking slanted nanograting. *Nat. Commun.* **15**, 3055 (2024).

36. T. Norden, L. M. Martinez, N. Tarefder, K. W. C. Kwock, L. M. McClintock, N. Olsen, L. N. Holtzman, J. H. Yeo, L. Zhao, X. Zhu, J. C. Hone, J. Yoo, J.-X. Zhu, P. J. Schuck, A. J. Taylor, R. P. Prasankumar, W. J. M. Kort-Kamp, P. Padmanabhan, Twisted nonlinear optics in monolayer van der Waals crystals. *ACS Nano* **19**, 30919–30929 (2025).

37. F. J. F. Loechner, R. Mupparapu, M. Steinert, A. George, Z. Tang, A. Turchanin, T. Pertsch, I. Staude, F. Setzpfandt, Controlling second-harmonic diffraction by nano-patterning MoS_2 monolayers. *Opt. Express* **27**, 35475–35484 (2019).

38. J. Deka, J. Gao, X. Yang, Anisotropic third-harmonic vortex beam generation with ultrathin germanium arsenide fork gratings. *Laser Photonics Rev.* **19**, 2401519 (2025).

39. E. A. Fajardo, R. Winkler, Effective dynamics of two-dimensional Bloch electrons in external fields derived from symmetry. *Phys. Rev. B* **100**, 125301 (2019).

40. R. W. Boyd, Nonlinear Optics (Academic Press, 2020).

41. D. Zhang, Z. Zeng, Q. Tong, Y. Jiang, S. Chen, B. Zheng, J. Qu, F. Li, W. Zheng, F. Jiang, H. Zhao, L. Huang, K. Braun, A. J. Meixner, X. Wang, A. Pan, Near-unity polarization of valley-dependent second-harmonic generation in stacked TMDC layers and heterostructures at room temperature. *Adv. Mater.* **32**, e1908061 (2020).

42. A. Dasgupta, J. Gao, X. Yang, Second-harmonic optical vortex conversion from WS_2 monolayer. *Sci. Rep.* **9**, 8780 (2019)

43. E. Menshikov, P. Franceschini, K. Frizyuk, I. Fernandez-Corbaton, A. Tognazzi, A. C. Cino, D. Garoli, M. Petrov, D. de Ceglia, C. De Angelis, Light structuring via nonlinear total angular momentum addition with flat optics. *Light Sci. Appl.* **14**, 381 (2025).

44. N. Kumar, S. S. G. Buddha, P. Choudhury, K. S. Malik, A. S. Deo, Computer generated holography: Techniques, applications, and future directions, in *Materials for Electronic, Magnetic, and Spintronic Technologies: Characterization and Applications from Energy Storage to Disease Detection*, A. Mishra, V. Dixit, D. Somvanshi, A. Singh, A. Mishra, Eds. (Springer Nature Switzerland, Cham, 2024), pp. 153–169.

45. A. Shapira, I. Juwiler, A. Arie, Nonlinear computer-generated holograms. *Opt. Lett.* **36**, 3015–3017 (2011).

46. Z. Xie, T. Zhao, X. Yu, J. Wang, Nonlinear optical properties of 2D materials and their applications. *Small* **20**, e2311621 (2024).

47. Y. Sheng, Q. Kong, W. Wang, K. Kalinowski, W. Krolikowski, Theoretical investigations of nonlinear Raman–Nath diffraction in the frequency doubling process. *J. Phys. B At. Mol. Opt. Phys.* **45**, 055401 (2012).

48. K. F. Mak, C. Lee, J. Hone, J. Shan, T. F. Heinz, Atomically thin MoS_2: A new direct-gap semiconductor. *Phys. Rev. Lett.* **105**, 136805 (2010).

49. S. Klimmer, T. Lettau, L. Valencia Molina, D. Kartashov, U. Peschel, J. Wilhelm, D. N. Neshev, G. Soavi, Probing ultrafast coherent bandgap modulation in monolayer WSe_2 by nonlinear optics. *Adv. Opt. Mater.* **14**, e03236 (2025).

50. J. C. T. Lee, S. J. Alexander, S. D. Kevan, S. Roy, B. J. McMorran, Laguerre–Gauss and Hermite–Gauss soft X-ray states generated using diffractive optics. *Nat. Photonics* **13**, 205–209 (2019).

51. X. Fang, H. Ren, M. Gu, Orbital angular momentum holography for high-security encryption. *Nat. Photonics* **14**, 102–108 (2020).

52. Z. Zhang, X. Qiao, B. Midya, K. Liu, J. Sun, T. Wu, W. Liu, R. Agarwal, J. M. Jornet, S. Longhi, N. M. Litchinitser, L. Feng, Tunable topological charge vortex microlaser. *Science* **368**, 760–763 (2020).

53. J. Wang, Advances in communications using optical vortices. *Photonics Res.* **4**, B14 (2016).

54. X. Wang, Y. Liu, L. Guo, H. Li, Potential of vortex beams with orbital angular momentum modulation for deep-space optical communication. *Opt. Eng.* **53**, 056107 (2014).

55. F. Friedrich, P. Herrmann, S. S. Shanbhag, S. Klimmer, J. Wilhelm, G. Soavi, Measurement of optically induced broken time-reversal symmetry in atomically thin crystals. *Nat. Photonics* **20**, 186–193 (2026).

56. J. Ni, C. Huang, L.-M. Zhou, M. Gu, Q. Song, Y. Kivshar, C.-W. Qiu, Multidimensional phase singularities in nanophotonics. *Science* **374**, eabj0039 (2021).

57. X. Qiao, B. Midya, Z. Gao, Z. Zhang, H. Zhao, T. Wu, J. Yim, R. Agarwal, N. M. Litchinitser, L. Feng, Higher-dimensional supersymmetric microlaser arrays. *Science* **372**, 403–408 (2021).

58. K. L. Seyler, J. R. Schaibley, P. Gong, P. Rivera, A. M. Jones, S. Wu, J. Yan, D. G. Mandrus, W. Yao, X. Xu, Electrical control of second-harmonic generation in a WSe_2 monolayer transistor. *Nat. Nanotechnol.* **10**, 407–411 (2015).

59. W. Liu, Z. Ji, Y. Wang, G. Modi, M. Hwang, B. Zheng, V. J. Sorger, A. Pan, R. Agarwal, Generation of helical topological exciton-polaritons. *Science* **370**, 600–604 (2020).
